# Folic Acid-Guided PLGA-Zein Core–Shell Nanoparticles for Co-Delivery of Temozolomide and Ellagic Acid to Overcome PARP-Mediated Chemoresistance in Glioblastoma

**DOI:** 10.3390/pharmaceutics18060655

**Published:** 2026-05-27

**Authors:** Arunraj Tharamelveliyil Rajendran, Ashwini Prabhu, Ashwini Madhava, Anoop Narayanan Vadakkepushpakath

**Affiliations:** 1 Nitte (Deemed to be University), NGSM Institute of Pharmaceutical Sciences (NGSMIPS), Department of Pharmaceutics, Mangalore 575018, Karnataka, India; arunraj.21phdp102@student.nitte.edu.in (A.T.R.); ashwini.m@nitte.edu.in (A.M.); 2 Division of Cancer Research and Therapeutics (CaRT), Yenepoya Research Centre, Yenepoya (Deemed to be University), Mangalore 575018, Karnataka, India; ashwiniprabhu@srinivasuniversity.edu.in; 3 Central Research Laboratory, Srinivas Institute of Medical Sciences and Research Centre, Srinivas University, Mangalore 574146, Karnataka, India

**Keywords:** core–shell nanoparticles, PARP, glioblastoma, temozolomide, ellagic acid

## Abstract

**Background:** Glioblastoma (GBM) remains a lethal malignancy due to temozolomide (TMZ) resistance and limited drug penetration across the blood–brain barrier, largely driven by hyperactive DNA damage repair mechanisms such as poly (ADP-ribose) polymerase (PARP). To address these challenges, we developed folic acid-targeted PLGA–zein hybrid core–shell nanoparticles for the codelivery of the alkylating agent TMZ and the natural PARP inhibitor Ellagic acid (FA-TMZ/EA-PZ-CS NPs), thereby enabling simultaneous enhancement of drug delivery and suppression of chemoresistance pathways. **Methods and Results:** The dual-drug nanoplatform was fabricated using a double-emulsion solvent evaporation method and functionalized via EDC/NHS-mediated folic acid conjugation to promote receptor-mediated uptake. Physicochemical characterisation confirmed uniform spherical morphology, high colloidal stability, efficient drug encapsulation, and sustained biphasic drug release consistent with a core–shell diffusion mechanism. In LN229 glioblastoma cells, folic acid conjugation significantly enhanced cellular internalisation and cytotoxic efficacy compared to free drugs and non-targeted nanoparticles. Combination index analysis revealed strong synergism between TMZ and ellagic acid, resulting in markedly reduced IC_50_ values. Mechanistic studies demonstrated apoptosis induction, increased DNA damage, inhibition of cell migration at sub-cytotoxic concentrations, and downregulation of PARP gene expression. **Conclusion:** Overall, this study establishes a targeted core–shell nanotherapeutic strategy that integrates chemotherapy with DNA repair inhibition to overcome TMZ resistance, offering a mechanistically sound strategy that serves as a foundational framework for future translational research.

## 1. Introduction

Glioblastoma (GBM) is the most aggressive primary malignant brain tumour in adults, with a median overall survival of approximately 15–18 months despite standard treatment consisting of maximal surgical resection followed by radiotherapy and temozolomide (TMZ)-based chemotherapy, and fewer than 10% of patients surviving beyond five years [1]. The persistence of such poor outcomes is driven by several converging challenges: the blood–brain barrier (BBB), which severely restricts therapeutic delivery to the tumour site; rapid tumour cell proliferation; intra-tumoural heterogeneity; and the presence of GBM stem cells (GSCs), which sustain recurrence and confer resistance to therapy [2,3]. Temozolomide remains the frontline chemotherapeutic agent for GBM because of its oral bioavailability and ability to induce DNA alkylation-mediated cytotoxicity. However, its clinical efficacy is frequently compromised by intrinsic and acquired resistance mechanisms, particularly O^6^-methylguanine-DNA methyltransferase (MGMT)-mediated DNA repair and activation of alternative DNA damage response pathways. Therefore, combination strategies capable of sensitizing GBM cells to TMZ while suppressing repair mechanisms are considered promising approaches for improving therapeutic outcomes [4,5].

Poly (ADP-ribose) polymerase (PARP) inhibitors represent a rational combinatorial strategy to sensitise GBM cells to TMZ. PARP enzymes are central to base excision repair (BER), a DNA damage repair pathway that GBM cells exploit following TMZ-induced alkylation. Inhibiting PARP blocks this repair mechanism, amplifying DNA strand breaks and promoting tumour cell apoptosis, particularly in tumours with homologous recombination deficiencies [6,7]. However, most clinically used PARP inhibitors face major pharmacological barriers in GBM, including poor BBB penetration due to efflux by ABC transporters, as well as systemic toxicity at therapeutic doses [8,9].

Ellagic acid (EA), a naturally occurring polyphenol abundant in fruits and nuts, has emerged as a compelling plant-derived PARP inhibitor with additional antioxidant, anti-inflammatory, and antiproliferative properties [4,10]. Its multi-modal mechanism of action and favourable safety profile make EA an attractive alternative or adjunct to synthetic PARP inhibitors. Nevertheless, EA suffers from poor aqueous solubility, limited bioavailability, and insufficient BBB penetration, which constrain its clinical utility when administered in free form.

Nanoparticle-based delivery systems offer a transformative approach to overcoming these barriers. In particular, core–shell architectures, comprising a drug-loaded polymeric core surrounded by a functional shell, enable co-encapsulation of multiple therapeutic agents, controlled and sustained drug release, and surface modification for active tumour targeting [11]. Poly(lactic-co-glycolic acid) (PLGA), an FDA-approved biodegradable polymer, is widely employed as the core material due to its ability to encapsulate both hydrophilic and hydrophobic drugs and provide sustained release profiles that reduce systemic toxicity [12]. Zein, a plant-derived corn protein, complements PLGA as a shell material: its self-assembling properties, abundant surface functional groups, and biocompatibility make it highly suitable for drug loading modulation, release kinetics control, and ligand conjugation [13]. Active tumour targeting is achieved through conjugation of folic acid (FA) to the nanoparticle surface. The folate receptor-α (FR-α) isoform is overexpressed on GBM cells while remaining minimally expressed in normal brain tissue, enabling receptor-mediated endocytosis and selective intracellular delivery of the therapeutic payload while minimising off-target effects [14].

**Figure 1:**
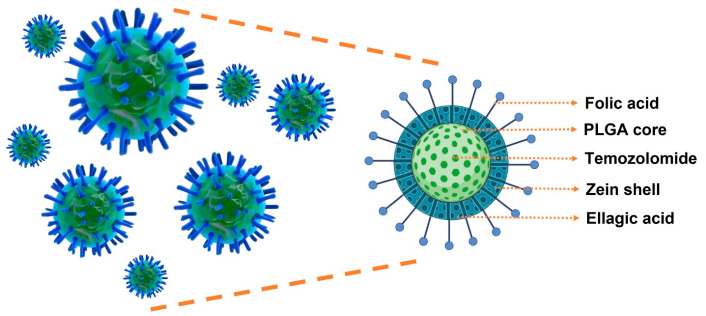
Structure of the folic acid-conjugated PLGA-Zein core–shell nanostructure loaded with dual drugs.

In this study, we developed a novel folic acid-conjugated PLGA–zein core–shell nanoparticle system for the co-delivery of temozolomide and ellagic acid against glioblastoma (Figure 1). Temozolomide was encapsulated within the PLGA core to provide sustained intracellular delivery, while ellagic acid was incorporated into the zein shell to enhance loading and rapid therapeutic availability. Surface-conjugated folic acid was introduced to promote folate receptor-mediated uptake in GBM cells. While folic acid-targeted systems are known, the unique contribution of this work is the structural integration of a standard alkylating agent and a natural PARP-inhibitor within a hierarchical protein–polymer hybrid platform. TMZ induces DNA damage, whereas EA acts as a natural PARP-inhibitory chemosensitizer that suppresses DNA repair and amplifies apoptotic signalling. To the best of our knowledge, this is among the first reports describing a folate-targeted PLGA–zein core–shell nanosystem integrating chemotherapy with natural PARP inhibition for glioblastoma therapy. The developed formulation was comprehensively evaluated for physicochemical characteristics, pH responsive drug release, cellular internalization, spheroid penetration, and in vitro anticancer efficacy.

## 2. Materials and Methods

### 2.1. Materials

Zein was purchased from Tokyo Chemical Industry Co., Ltd., Tokyo, Japan. Ellagic acid was procured from BLD Pharmatech (India) Pvt Ltd., Hyderabad, India. Folic acid was purchased from Chempure Pvt Ltd., Bangalore, India. Temozolomide was obtained from BLD Pharma Tech (India) Pvt Ltd. PLGA (75/25), 1-(3-dimethylaminopropyl)-3-ethylcarbodiimide hydrochloride (EDC), N-hydroxy succinimide (NHS), and Dimethyl sulfoxide (DMSO) were purchased from Sigma-Aldrich (St. Louis, MO, USA). Acetone and methanol were acquired from Loba Chemie Pvt Ltd. (Mumbai, India), while ethanol was supplied by Changshu Hongsheng Fine Chemical Co. Ltd. (Changshu, China). Dulbecco’s Modified Eagle Medium (DMEM), Fetal Bovine Serum (FBS), antibiotic-antimycotic solution, and Trypsin-EDTA solution were obtained from Gibco (Life Technologies AG, Basel, Switzerland). MTT (3-(4,5-Dimethylthiazol-2-yl)-2,5-Diphenyltetrazolium Bromide), Crystal Violet, Fluorescein Isothiocyanate, and Agarose Low EEO were procured from Himedia, Maharashtra, India. Acridine Orange, Ethidium Bromide, Rhodamine B, Hoechst 33342, Trypan Blue, Propidium iodide and Phosphate-buffered saline (PBS) for cell culture without calcium and magnesium were purchased from Invitrogen (Waltham, MA, USA). GENLISA™. Human Poly ADP Ribose Polymerase (PARP) ELISA kit was purchased from Krishgen Biosystems Private Limited, Mumbai, India. Glioblastoma LN229 cells and L929 fibroblast cells were procured from the Cell repository, National Centre for Cell Science (NCCS), Pune, India. Human PARP Forward Primer (5′ to 3′) CCAAGCCAGTTCAGGACCTCAT, Human PARP Reverse Primer (3′ to 5′) GGATCTGCCTTTTGCTCAGCTTC, Human Beta Actin Forward Primer (5′ to 3′) CACCATTGGCAATGAGCGGTTC, Human Beta Actin Reverse Primer (3′ to 5′) AGGTCTTTGCGGATGTCCACGT were procured from Sigma-Aldrich. All chemicals and reagents used in this study were of analytical, HPLC, or laboratory grade, with a purity greater than 95%.

### 2.2. Preparation of Plain and Drug-Loaded PLGA–Zein Core–Shell Nanoparticles

Plain PLGA–zein core–shell nanoparticles were fabricated using a controlled two-step nanoprecipitation approach. In the first step, PLGA (20 mg) was dissolved in 10 mL acetone (2 mg/mL) to form the organic phase, and this solution was added dropwise into 20 mL of ultrapure water under probe sonication with 30% amplitude for 5 min in pulse mode (5 s ON/3 s OFF) under ice-cold conditions. Rapid diffusion of acetone into the aqueous phase resulted in spontaneous precipitation of PLGA nanoparticles. The nanosuspension was further stirred at room temperature for 30 min to allow complete solvent evaporation and particle stabilization. In the second step, zein (20 mg) was dissolved in 10 mL of 70% *v*/*v* ethanol (2 mg/mL) under magnetic stirring until a clear solution was obtained. The zein solution was then added dropwise to the preformed PLGA nanosuspension under continuous stirring (700 rpm). After complete addition, the pH was gradually reduced to 4.0 using 1 N HCl under gentle stirring. The decrease in pH induced desolvation and deposition of zein around the PLGA nanoparticles, forming a uniform protein shell. Stirring was continued for an additional 1 h to ensure complete shell formation. The resulting nanoparticles were collected through repeated centrifugation (12,000 rpm, 10 min; Avanti J26 XP, Beckman Coulter, Brea, CA, USA) and washed to remove unbound components before lyophilization. For the preparation of the dual drug-loaded nanosystem, the same two-step procedure was followed, with drug incorporation aligned to the core–shell architecture. TMZ was mixed with PLGA in acetone at defined TMZ: PLGA weight ratios (1:1, 1:2, 1:3, 1:4 and 1:5, *w*/*w*) prior to nanoprecipitation, enabling efficient entrapment within the hydrophobic PLGA core during polymer solidification. EA was incorporated during the shell formation stage by dissolving it in the zein ethanolic solution at EA: zein ratios of 1:1, 1:2, 1:3, 1:4, and 1:5 (*w*/*w*), promoting drug–protein association driven by π–π stacking and hydrophobic affinity. Following the addition of the EA–zein phase to the TMZ-loaded PLGA dispersion and controlled pH reduction with 1 N HCl, a compact EA-enriched zein shell was formed around the TMZ-loaded core. The dual-drug nanoparticles were purified by repeated centrifugation and washing, and the final optimised formulation was lyophilised for long-term stability and subsequent characterisation.

#### Folic Acid Conjugation via EDC/NHS Mechanism

Folic acid (FA) was conjugated onto the zein shell of the core–shell nanoparticles via EDC.HCl/NHS-mediated carbodiimide coupling. Briefly, 5 mg of FA was dispersed in 50 mL of 0.1 M MES buffer (pH 5.5) to obtain a concentration of 0.1 mg/mL, and the mixture was sonicated for 15 min to ensure uniform dispersion. The carboxyl groups of FA were activated by adding 5 mL each of EDC.HCl (1 mg/mL) and NHS (1 mg/mL), followed by sonication for 20 min. Optimized core–shell nanoparticles (80 mg), dispersed in 20 mL of distilled water, were then added to the activated FA solution and stirred continuously for 3 h at room temperature. The FA-conjugated nanoparticles were recovered by centrifugation (12,000 rpm, 15 min, 4 °C), washed three times with ultrapure water to remove unreacted EDC.HCl, NHS, and unbound FA, and lyophilised for subsequent characterisation. FT-IR and ^1^H NMR studies confirmed successful conjugation.

### 2.3. Characterisation of Plain and Dual-Drug-Loaded PLGA–Zein Core–Shell Nanoparticles

The physicochemical and structural characteristics of the formulated PLGA–zein core–shell nanoparticles were analysed using advanced techniques to evaluate their size distribution, surface charge, morphology, chemical interactions, molecular structure, crystallinity, and thermal behaviour. The hydrodynamic diameter, PDI and zeta potential of the nanoparticles were measured using a Malvern Zetasizer (Nano ZS, Malvern Instruments, Malvern, UK. The surface morphology and core–shell structure of the nanoparticles were examined using Transmission Electron Microscopy (TECNAI 12 G2), Hillsboro, OR, USA.

#### 2.3.1. Fourier Transform Infrared Spectroscopy (FT-IR)

FT-IR spectroscopy was used to analyse the functional groups and drug polymer interactions between the polymer and drugs. The spectra of plain PLGA, zein, temozolomide, ellagic acid, folic acid, plain PLGA–zein core–shell nanoparticles, PLGA-TMZ, zein-EA FA-zein, and FA-TMZ/EA-PZ-CS NPs were recorded using an FT-IR spectrometer (Bruker Alpha II, Billerica, MA, USA) over a wavelength range of 4000–400 cm^−1^.

#### 2.3.2. Proton Nuclear Magnetic Resonance Spectroscopy (^1^H-NMR)

^1^H-NMR spectroscopy was performed to verify the successful conjugation of folic acid on the zein NPs. The samples were dissolved in an appropriate deuterated solvent (DMSO-d_6_ and CDCl_3_), and spectra were recorded using a Fourier Transform NMR spectrometer (Avance Neo 400 MHz, Bruker, Fällanden, Switzerland). The characteristic peaks of zein, folic acid, and folic acid-conjugated zein NPs were analysed to confirm molecular interactions.

#### 2.3.3. X-Ray Diffraction (XRD) Analysis

The crystalline or amorphous nature of the nanoparticles was evaluated using X-ray Diffraction (XRD). Powdered samples were analysed using an X-ray diffractometer (Empyrean 3rd Gen, Malvern PANalytical, Almelo, Netherlands), operating at a Cu-Kα radiation source (λ = 1.5406 Å) over a 2θ range of 0–80 °C. The diffraction patterns of (A) zein, (B) PLGA (75:25), (C) TMZ, (D) EA, (E) Folic acid, and (F) FA-TMZ/EA-PZ-CS NPs were compared to determine changes in crystallinity, which could indicate successful encapsulation and interaction between the core and shell materials.

#### 2.3.4. Differential Scanning Calorimetry (DSC)

The thermal behaviour of the nanoparticles was analysed using a differential scanning calorimeter (DSC 60+, Shimadzu, Kyoto, Japan). The thermograms of (A) zein, (B) PLGA (75:25), (C) TMZ, (D) EA, (E) Folic acid, and (F) FA-TMZ/EA-PZ-CS NPs were recorded at heating rate: 10 °C/min; nitrogen atmosphere; temperature range: 25–400 °C used to study possible changes in melting temperature (Tm) and glass transition temperature (Tg), indicating nanoparticle formation and interactions between the core–shell materials.

#### 2.3.5. Simultaneous UV Estimation of Temozolomide and Ellagic Acid

Standard stock solutions of TMZ and EA were prepared by accurately weighing 10 mg of each compound, dissolving them in a suitable solvent, and making up the volume to 50 mL in separate volumetric flasks to obtain a concentration of 100 μg/mL. The solutions were sonicated to ensure complete dissolution, and subsequent working solutions were prepared by serial dilution to achieve concentration ranges of 3–18 μg/mL for TMZ and 1–9 μg/mL for EA. Millipore-grade water served as the analytical blank. For wavelength selection, individual standard solutions of both drugs were scanned in the UV–visible range of 200–400 nm using a UV/Vis spectrophotometer, Shimadzu Kyto, Japan. Calibration curves were plotted by measuring absorbance at the respective λ-max values, and linearity was confirmed across the selected concentration ranges. The concentration of each component can be calculated by mathematical Equation (1)

A1 = ax C1 + bx C2(1)

A2 = cx C1 + dx C2

where ax = absorptivity of ellagic acid at λ-max 277, bx = absorptivity of temozolomide at λ-max 277, cx = absorptivity of ellagic acid at λ-max 328, dx = absorptivity of temozolomide at λ-max 328, C1 = concentration of ellagic acid, and C2 = concentration of temozolomide, where A1 and A2 are the absorbance of the mixture sample at two different wavelengths (λ-max 277 and 328, respectively).

#### 2.3.6. Entrapment Efficiency and Drug Loading Content

The entrapment efficiency (EE) and drug loading content (DLC) of the formulated PLGA–zein core–shell nanoparticles were quantified using a validated UV–Vis spectrophotometric method based on the simultaneous equation approach (Cramer’s rule). The percentage of drug successfully encapsulated within the nanoparticles was determined by measuring the concentration of free (unencapsulated) drug in the supernatant after centrifugation. Briefly, a known quantity of drug-loaded nanoparticles was centrifuged at 15,000 rpm for 30 min at 4 °C, and the supernatant was collected. The unencapsulated drug content was quantified using UV–Vis Spectrophotometer (UV-1900i, Shimadzu, Kyoto, Japan. The EE% was calculated using the following Equation (2).

EE (%) = (Total drug added − Free drug in supernatant)/Total drug added × 100(2)

#### 2.3.7. Drug Loading Content (DLC%)

The amount of drug incorporated within the nanoparticles relative to the total weight of nanoparticles was determined by dissolving a known mass of lyophilized nanoparticles in an organic solvent (DMSO), followed by drug quantification. The DLC % was calculated using the following Equation (3).

DLC (%) = (Encapsulated drug/Total weight of the nanoparticles) × 100(3)

Both parameters were analysed in triplicate, and the results were reported as the mean ± standard deviation (SD). These values provide crucial insights into the formulation efficiency and drug-loading capacity of the developed core–shell nanoparticles.

#### 2.3.8. Drug Release Studies and Kinetics

The in vitro drug release profile of dual drug-loaded PLGA–zein core–shell nanoparticles was evaluated using the Pur-A-Lyzer dialysis method to simulate physiological conditions. A pre-treated Pur-A-Lyzer dialysis membrane (MWCO: 12–14 kDa) was employed to facilitate the diffusion of free drug molecules while retaining nanoparticles within the enclosed system. Briefly, a known quantity of nanoparticles (equivalent to a fixed amount of TMZ and EA) was dispersed in 2 mL of phosphate-buffered saline (PBS, pH 7.4) or acetate buffer (pH 5.5) and loaded into the dialysis membrane, which was subsequently immersed in 100 mL of release medium under continuous stirring (100 rpm) at 37 ± 0.5 °C. At predetermined time intervals (0.5, 1, 2, 4, 8, 12, 24, 48, and 72 h), aliquots (1 mL) were withdrawn and replenished with an equal volume of fresh medium to maintain sink conditions. The concentrations of TMZ and EA released into the medium were quantified via UV–Vis spectrophotometry (simultaneous estimation method) at their respective λmax values, and the cumulative release percentage was determined using Equation (4).

Cumulative drug release (%) = (Drug released at time t/Total drug encapsulated) × 100(4)

To elucidate the drug release kinetics and underlying mechanism, the release data were fitted into mathematical models, including zero-order (constant release), first-order (concentration-dependent release), Higuchi (diffusion-controlled release), and Korsmeyer–Peppas models (polymer matrix-based release). The correlation coefficient (R^2^ value) was used to determine the best-fit model.

### 2.4. In Vitro Hemocompatibility Assay

The hemocompatibility of the FA-TMZ/EA-PZ-CS NPs were assessed via hemolysis assay to ensure their safety for intravenous administration. Unidentified human blood was collected from the blood bank (CEC approval number: NU/CEC/2023/490, study approved date on 9 October 2023). A total of 0.1 mL of NP samples of PLGA–zein core–shell nanoparticles (25–1000 µg/mL) were incubated with human blood (0.9 mL) containing ACD for 3 h at 37 °C under shaking. The resulting plasma was collected by centrifugation at 4500 rpm for 10 min. A total of 0.1 mL of plasma sample was diluted with 0.9 mL of 0.1% sodium carbonate and analysed for the presence of plasma haemoglobin (Hb) using optical density measurements. The positive and negative controls in the current experiment involve saline and triton-X (1%) treated blood. Plasma haemoglobin concentration was quantified spectrophotometrically, which is directly proportional to the concentration of lysed blood cells, and can be directly correlated to the haemolytic activity of the material. The plasma Hb concentration and the percentage hemolysis values can be determined using the following Equation (5).

Amount of Plasma Hb = 2 × A_415_ − (A_380_ + A_450_) × 1000 × Dilution factor/(E × 1.655) (5)

where A_415_, A_380_, and A_450_ are the absorbance values at 415, 380, and 450 nm. A_415_ is the Soret band absorption of haemoglobin, while A_380_ and A_450_ are correction factors relevant to uroporphyrin absorbing in the same wavelength range. E is the molar absorptivity of oxyhaemoglobin at 415 nm, which is 79.46. The correction factor applied due to the turbidity of the plasma sample is 1.655. Triplicate samples were analysed, and the haemolytic property of nanoparticles was plotted as percentage hemolysis, calculated as following Equation (6).

Hemolysis (%) = Plasma Hb content in the test/Total Hb value of blood × 100(6)

#### 2.4.1. In Vitro Cell Studies: Cytocompatibility of FA-TMZ/EA-PZ-CS NPs

The in vitro cytocompatibility of FA-TMZ/EA-PZ-CS NPs was evaluated using L929 fibroblast cells (collected from the cell repository, National Centre for Cell Science, Pune, India) for biomaterial biocompatibility assessment. L929 cells were seeded in 96-well plates at a density of 5 × 10^3^ cells per well and incubated in DMEM supplemented with 10% fetal bovine serum (FBS) and 1% penicillin-streptomycin at 37 °C under 5% CO_2_. After 24 h, the cells were treated with varying concentrations of nanoparticles (5–1000 µg/mL) and then incubated for an additional 24 h. Cytocompatibility was evaluated using the MTT assay, where 100 µL of 1 mg/mL MTT solution was added to each well and incubated for 4 h. The formazan crystals were dissolved in DMSO (100 μL per well), and the absorbance was measured at 570 nm using a microplate reader. Cell viability was calculated relative to the untreated control.

#### 2.4.2. Quantitative Cytotoxicity Profiling of Temozolomide, Ellagic Acid, and Their Synergistic Combination in Glioma Cells

The cytotoxicity of TMZ, EA, and their combinations was assessed using the MTT assay on the GBM cell line (LN229) collected from the cell repository, National Centre for Cell Science, Pune. Cells were seeded in 96-well plates at a density of 5 × 10^4^ cells/well and allowed to adhere overnight. Stock solutions of TMZ and EA were prepared in DMSO and PBS, and serial dilutions were made to achieve a range of concentrations. Cells were treated with TMZ, EA, and combinations of TMZ + EA at fixed dose ratios. Treatment with TMZ and EA alone was carried out at the following doses: 10, 25, 100, and 250 μg/mL. For the combination, a 1:1 ratio was used. Accordingly, TMZ and EA were co-administered in the following paired concentrations (5 µg/mL + 5 µg/mL, 12.5 µg/mL + 12.5 µg/mL, 25 µg/mL + 25 µg/mL, 50 µg/mL + 50 µg/mL, and 125 µg/mL + 125 µg/mL). After 48 h of incubation, the MTT reagent (1 mg/mL) was added and incubated for an additional 4 h at 37 °C. The formazan crystals were formed and dissolved in DMSO, and the absorbance was measured at 570 nm using a microplate reader. % Cell viability was calculated, and dose–response curves were generated. All experiments were performed in three independent biological replicates (*n* = 3), with each treatment tested in triplicate wells within each experiment. The synergistic effect of TMZ and EA in GBM therapy was evaluated using the Chou–Talalay method (CompuSyn software) version 3.0.1, a widely accepted approach for determining drug interactions based on the median-effect principle. The combination index (CI) values were calculated to quantify the nature of drug interactions, where CI < 1 indicates synergy, CI = 1 indicates an additive effect, and CI > 1 indicates antagonism. The resulting dose–response data were used to generate Fa–log(D) plots, where Fa represents the fraction of cells affected (cell death) and ranges from 0 (no effect) to 1 (100% cell death).

CI = (D)1/(Dx) 1 + (D)2/(Dx) 2(7)

where (D)_1_ and (D)_2_ are the doses of TMZ and EA in combination, required to achieve a certain level of inhibition (Fa, fraction affected), and (D_x_)_1_ and (D_x_)_2_ are the doses of each drug alone, required to achieve the same effect. CI values were calculated for different Fa levels (Fa = 0.25, 0.5, 0.75, and 0.9), corresponding to 25%, 50%, 75%, and 90% inhibition, respectively.

#### 2.4.3. Cytotoxicity Evaluation of Non-Conjugated (TMZ/EA-PZ-CS NPs) and Folic Acid-Conjugated Dual Drug-Loaded PLGA–Zein Core–Shell Nanoparticles (FA-TMZ/EA-PZ-CS NPs)

The cytotoxic potential of non-conjugated and folic acid-conjugated dual drug-loaded PLGA–zein core–shell nanoparticles was evaluated in LN229 glioblastoma cells and compared with bare TMZ, bare EA, and their combination (TMZ + EA) using the MTT assay. LN229 cells were cultured in DMEM supplemented with 10% FBS and 1% penicillin-streptomycin and maintained at 37 °C in a 5% CO_2_ incubator. Cells were seeded at a density of 5 × 10^4^ cells per well in a 96-well plate and allowed to adhere for 24 h before treatment. Serial dilutions of bare TMZ, bare EA, and FA-conjugated nanoparticles (equivalent drug concentrations) were prepared in complete DMEM, and 100 µL of each formulation was added to the respective wells. Control wells included untreated cells and vehicle-treated cells (DMSO < 0.1%). After 48 h of incubation, cell viability was assessed using the MTT assay, in which 100 µL of 1 mg/mL MTT solution was added to each well and incubated for 4 h at 37 °C. The formed formazan crystals were solubilised with 100 µL of DMSO, and the absorbance was measured at 570 nm using a microplate reader. Cell viability was calculated relative to the untreated control, and dose–response curves were generated to determine the IC_50_ values for each treatment group. All experiments were conducted in three independent biological replicates (n = 3), with each treatment tested in triplicate wells within each experiment. Following IC_50_ determination, the equivalent cytotoxic doses were chosen as the working concentrations for downstream in vitro assays.

#### 2.4.4. Cellular Uptake Studies

Cellular internalization of TMZ/EA-PZ-CS NPs and FA-TMZ/EA-PZ-CS NPs was carried out using fluorescence microscopy, Bio-Rad, Hercules, CA, USA. Rhod 123 was labelled to the polymer–protein core–shell NPs using ionic interaction and physical adsorption. The redispersed nanoformulations were separately conjugated with Rhodamine B (5 mg/mL, 0.5 mL) in a dark condition and stirred overnight. The resulting Rhod 123 labelled nanoparticles were centrifuged (15,000 rpm for 15 min) and washed (3 times with water). The washed pellet was redispersed in saline and used for internalization studies. LN229 cells were seeded in 24-well plates with a density of 20,000 cells/well. Once the cells were attached, the medium was removed, and the cells were washed with PBS. They were then incubated with Rhod 123-labelled samples at a concentration of 1 mg/mL for 6 h. Fluorescence imaging was performed using a Zoe fluorescence imager (Bio-Rad, Hercules, CA, USA) with images acquired at 20× magnification to assess cellular uptake and intracellular localization.

#### 2.4.5. Trypan Blue Dye Exclusion Assay

The Trypan Blue dye exclusion assay was performed to assess the cytotoxic effects of TMZ/EA-PZ-CS NPs and FA-TMZ/EA-PZ-CS NPs on LN229 glioblastoma cells. LN229 cells were seeded in six-well plates at a density of 1 × 10^5^ cells per well and incubated overnight in DMEM supplemented with 10% FBS and 1% penicillin-streptomycin at 37 °C in a 5% CO_2_ atmosphere. Cells were then treated with IC_50_ concentrations of nanoparticles for 48 h. Following treatment, both adherent and floating cells were collected, washed with phosphate-buffered saline (PBS), and resuspended in 100 µL of PBS. An equal volume of 0.4% Trypan Blue solution was added, and the mixture was incubated for 2 min at room temperature. The number of viable (unstained) and non-viable (blue-stained) cells was counted using a haemocytometer under a light microscope.

Percentage of viable cells = Number of viable cells/Total number of cells × 100(8)

#### 2.4.6. Clonogenic Assay

A clonogenic survival assay was performed to assess the long-term proliferative potential of LN229 glioblastoma cells following treatment with TMZ/EA-PZ-CS NPs and HA-TMZ/NG-CS-TG CS NPs. LN229 cells were seeded in six-well plates at a low density of 500 cells per well and allowed to attach for 24 h under standard culture conditions (37 °C, 5% CO_2_, complete DMEM). Cells were then treated with IC_50_-based concentrations of the respective nanoparticle formulations for 48 h. After this, the treatment medium was replaced with drug-free DMEM to allow for colony formation over 10–14 days. Colonies were then fixed with 4% paraformaldehyde, stained with 0.5% crystal violet, and manually counted. The surviving fraction was determined relative to untreated control cells.

#### 2.4.7. Hoechst Staining

Hoechst 33342 staining was performed to evaluate nuclear morphology changes in LN229 glioblastoma cells following treatment with TMZ/EA-PZ-CS NPs, FA-TMZ/EA-PZ-CS NPs and compared with untreated cells. LN229 cells were seeded in 24-well plates at a density of 1 × 10^4^ cells per well and incubated overnight in DMEM supplemented with 10% FBS and 1% penicillin-streptomycin at 37 °C in a 5% CO_2_ atmosphere. Cells were then treated with IC_50_ concentrations of nanoformulations for 48 h. Afterwards, they were washed with phosphate-buffered saline (PBS) and fixed with methanol for 15 min at room temperature. Fixed cells were stained using 5 µg/mL Hoechst 33342 dye for 10 min in the dark. After washing with PBS, cells were visualized under a fluorescence microscope under the blue channel. Nuclear changes were identified by their characteristic features, including condensation, fragmentation, and increased fluorescence intensity, compared to untreated control cells.

#### 2.4.8. Live/Dead Assay (Acridine Orange/Ethidium Bromide (AO/EB) Staining Assay)

Acridine orange/ethidium bromide (AO/EB) dual staining was performed to assess apoptosis induction in LN229 glioblastoma cells following treatment with TMZ/EA-PZ-CS NPs and TMZ/EA-PZ-CS NPs. LN229 cells were seeded in 24-well plates at a density of 1 × 10^4^ cells per well and cultured overnight in DMEM supplemented with 10% FBS and 1% penicillin-streptomycin at 37 °C in a 5% CO_2_ incubator. Cells were then treated with IC_50_ dose concentrations of nanoparticles for 48 h, followed by washing with phosphate-buffered saline (PBS). Subsequently, 500 µL of AO/EB staining solution (1:1 mixture of 2 µg/mL acridine orange and ethidium bromide) was added to the cells and incubated for 5 min in the dark at 37 °C. The stained cells were immediately visualized under a fluorescence microscope. Viable cells exhibited green fluorescence with intact nuclei. Early apoptotic cells displayed yellow-green nuclei with condensation or fragmentation. Late apoptotic cells showed orange to red nuclei with condensed chromatin, and necrotic cells appeared uniformly red.

#### 2.4.9. Quantitative Validation of PARP-1 Protein Expression by ELISA

The quantitative determination of PARP-1 protein levels in LN229 glioblastoma cells following treatment with TMZ/EA-PZ-CS NPs and FA-TMZ/EA-PZ-CS NPs was performed to validate mechanistic claims at the protein level. This analysis was conducted using the high-sensitivity Krishgen’s GENLISA™ Human Poly Adp Ribose Polymerase (PARP) ELISA kit, Mubai, India which utilizes a sandwich ELISA technique for absolute protein quantification. Monoclonal antibodies are pre-coated onto microwell samples, and standards are pipetted into the microwells; the antibodies then bind to the human PARP present in the sample. The biotin-labelled antibody is added, followed by the addition of streptavidin-HRP conjugate, and the mixture is incubated to form a complex. After washing the microwells to remove any non-specific binding, the substrate solution (TMB) was added to the microwells, and the colour developed is directly proportional to the amount of Human PARP in the sample. The reaction was stopped with the addition of a stop solution. Absorbance was measured at 450 nm using a microplate reader.

#### 2.4.10. Gene Expression Analysis Using qRT-PCR

LN229 glioblastoma cells were seeded in six-well plates at a density of 5 × 10^4^ cells per well and cultured in complete DMEM at 37 °C in a humidified atmosphere with 5% CO_2_. After cell adherence, they were treated with TMZ/EA-PZ-CS NPs and FA-TMZ/EA-PZ-CS NPs at their respective IC_50_ concentrations and incubated for 48 h. Total RNA was isolated using TRI-Reagent. The purity and concentration of RNA were assessed using Nanodrop Spectrophotometer, Thermo Fisher Scientific, Wilmington, NC, USA, and samples with acceptable A_260_/A_280_ ratios were used for the qRT-PCR. Complementary DNA (cDNA) was synthesized using 1 µg of total RNA using a reverse transcription kit. Quantitative real-time PCR was performed using SYBR Green master mix in a real-time PCR system (CFX 96, BioRad, Hercules, CA, USA). PARP-specific primers (Human PARP Forward Primer (5′ to 3′) CCAAGCCAGTTCAGGACCTCAT, Human PARP Reverse Primer (3′ to 5′) GGATCTGCCTTTTGCTCAGCTTC, Human Beta Actin Forward Primer (5′ to 3′) CACCATTGGCAATGAGCGGTTC, Human Beta Actin Reverse Primer (3′ to 5′) AGGTCTTTGCGGATGTCCACGT). were used for amplification, with β-Actin as a housekeeping gene. Relative gene expression levels were quantified using the comparative 2^−ΔΔCt^ method and expressed as fold change relative to untreated control cells. Each reaction was carried out in triplicate and values were represented as Mean ± SD.

#### 2.4.11. Comet Assay

The Comet assay was performed to evaluate DNA damage in LN229 glioblastoma cells following treatment with IC_50_ concentrations of TMZ/EA-PZ-CS NPs and FA-TMZ/EA-PZ-CS NPs. LN229 cells were seeded in six-well plates at a density of 1 × 10^5^ cells per well and incubated overnight in DMEM supplemented with 10% FBS and 1% penicillin-streptomycin at 37 °C in a 5% CO_2_ incubator. Cells were then treated with IC_50_ concentrations of both nanoformulations for 48 h, harvested by trypsinization, and resuspended in phosphate-buffered saline (PBS). The cell suspension was mixed with 0.5% low-melting agarose and layered onto slides precoated with 0.6% normal-melting agarose, then solidified at 4 °C. The slides were immersed in lysis buffer (pH 10, containing 2.5 M NaCl, 100 mM EDTA, 10 mM Tris, 1% Triton X-100, and 10% DMSO) for 1 h at 4 °C to remove cellular membranes and proteins. Electrophoresis was performed under alkaline conditions (pH > 13, 300 mM NaOH, 1 mM EDTA) at 25 V, 300 mA for 20 min, followed by neutralization with 0.4 M Tris (pH 7.5) and staining with ethidium bromide (20 µg/mL). The slides were visualized under a fluorescence microscope, and DNA damage was evaluated.

#### 2.4.12. Scratch Assay

The scratch assay was performed to evaluate the effect of TMZ/EA-PZ-CS NPs and FA-TMZ/EA-PZ-CS NPs on the migration potential of LN229 glioblastoma cells. LN229 cells were seeded in six-well plates at a density of 5 × 10^5^ cells per well and cultured in DMEM supplemented with 10% FBS and 1% penicillin-streptomycin at 37 °C in a 5% CO_2_ incubator until a confluent monolayer was formed. A uniform scratch was created using a sterile 200-µL pipette tip, and detached cells were removed by washing with phosphate-buffered saline (PBS). Cells were then treated with serum-free DMEM containing nanoformulations at their respective IC_50_ concentrations. Untreated control wells received only serum-free DMEM. Wound closure was monitored at 0, 12, and 24 h using an inverted phase-contrast microscope, and images were captured for analysis. The percentage of wound closure was calculated using ImageJ software version 1.8.0, with the formula:

Wound closure (%) = (Initial wound area − Final wound area)/Initial wound area × 100(9)

## 3. Results

### 3.1. Development of Core–Shell Nanoparticles and Characterisation

**Figure 2:**
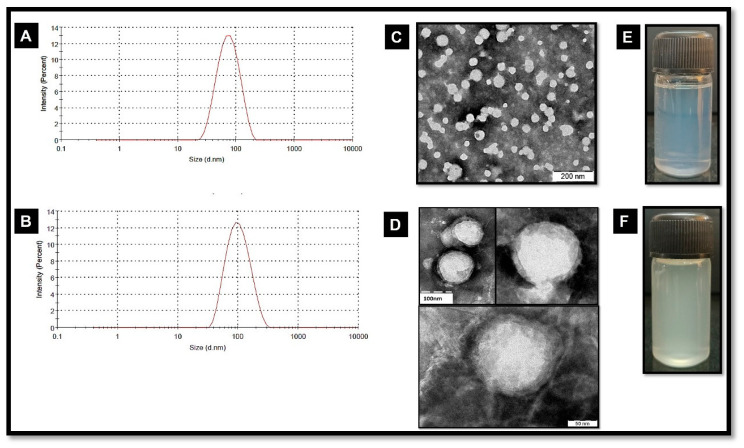
(**A**,**B**) shows the DLS pattern of plain PLGA and PLGA–Zein Core–Shell NPs. (**C**,**D**) illustrates TEM images of plain PLGA and PLGA–zein Core–Shell NPs. (**E**,**F**) represents the nano-formulation of plain PLGA and PLGA–zein Core–Shell NPs.

PLGA core nanoparticles were successfully prepared using the solvent evaporation method. Dynamic light scattering (DLS) analysis confirmed the successful formation of nanoscale particles with a controlled size distribution (Figure 2A), revealing a mean hydrodynamic diameter of 67.30 ± 8.2 nm and a polydispersity index (PDI) of 0.140, indicating a moderately narrow particle size distribution with good uniformity. The zeta potential of bare PLGA nanoparticles was measured at −28.2 mV, attributed to the presence of carboxyl end groups on the PLGA polymer chains. The core–shell architecture was achieved through an interfacial deposition technique where zein protein was deposited onto preformed PLGA cores. Following the zein coating, the hydrodynamic diameter increased to 91.50 ± 11.4 nm (Figure 2B), representing an approximately 30 nm increase in size, consistent with the formation of a protein shell layer. The PDI remained acceptably low at 0.162, demonstrating maintained colloidal homogeneity and acceptable size distribution. A significant shift in zeta potential to −38 mV was observed, indicating successful surface modification with zein, which possesses abundant ionizable amino acid residues. In contrast, the dual drug-loaded core–shell nanoparticles exhibited a slight increase in particle size to 190 ± 20 nm, which can be attributed to the incorporation of both drugs within the PLGA core and zein shell. Despite this increase, the PDI remained below 0.25, confirming that drug loading did not compromise particle stability or homogeneity. FA was conjugated to the zein shell surface through carbodiimide-mediated coupling chemistry using EDC/NHS activation. Following FA conjugation, the hydrodynamic diameter is slightly increased to 206 ± 8.2 nm, while the zeta potential became more negative due to the carboxyl group of folic acid. Figure S1 represents DLS and zeta potential pattern of FA-TMZ/EA-PZ-CS NPs. Table S1 illustrates the summary of particle size, polydispersity index (PDI), zeta potential, drug loading content, and entrapment efficiency of developed PLGA-based nanoparticle formulations. TEM images primarily confirmed the spherical morphology and core–shell architecture of the nanoparticles. The particle dimensions observed from TEM micrographs were in general agreement with DLS measurements, although slightly smaller apparent sizes are expected in TEM due to dehydration during sample preparation, whereas DLS measures hydrodynamic diameter in suspension. The plain PLGA nanoparticles (Figure 2C) appeared as distinct, well-defined spherical particles with smooth and uniform boundaries, exhibiting a consistent size distribution with no observable aggregation or structural collapse. The internal contrast of these particles suggested a solid polymeric structure characteristic of solvent-evaporated PLGA systems. In contrast, the plain PLGA–zein core–shell nanoparticles demonstrated (Figure 2D) a clear multilayer morphology, revealing a denser, darker inner core corresponding to the PLGA polymer, surrounded by a lighter, electron-transparent peripheral layer representing the zein coating. This contrast difference confirmed successful deposition of the zein shell around the PLGA core. The particles retained spherical geometry comparable to the plain PLGA nanoparticles; however, a slight increase in diameter was visually evident due to the additional shell layer. No particle fusion, deformation, or collapse was observed, indicating structural stability and efficient coating during the fabrication process. Figure 2E,F represents the images of the synthesized nanoparticle dispersions. (2E) Plain PLGA nanoparticles exhibiting a homogeneous, translucent suspension. (2F) PLGA–zein core–shell nanoparticles showing a stable and well-dispersed colloidal system with no visible precipitation, confirming successful formulation. The drug loading content of the dual-loaded PLGA–zein core–shell nanoparticles was calculated based on the theoretical mass of the formulation. The drug loading content was found to be 23.7% for TMZ and 23.4% for EA, corresponding to entrapment efficiencies of 94.7 ± 3.8% and 93.7 ± 4.2% (n = 3), respectively.

**Figure 3:**
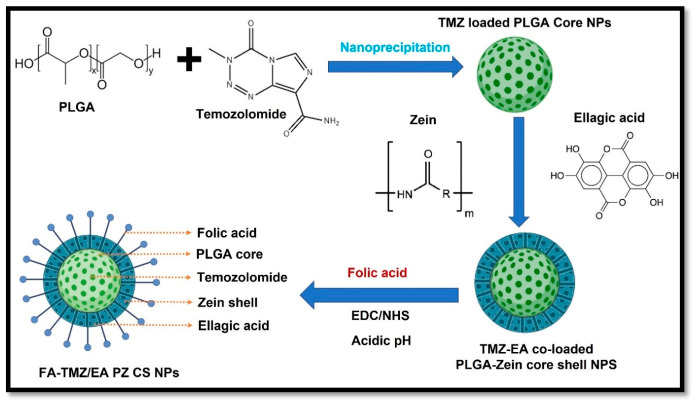
Schematic representation of the preparation of FA-TMZ/EA PZ CS NPs.

The UV–Visible absorption spectra of EA and TMZ were recorded in DMSO using DMSO as the reference blank to correct for background solvent absorption. As shown in Figure S2 EA and TMZ exhibited distinct absorption maxima at 277 nm and 328 nm, respectively. Calibration curves (Figure S3) for EA and TMZ were constructed over the tested concentration range in DMSO. Both drugs exhibited excellent linearity, with correlation coefficients (R^2^) of 0.9999 for EA and 0.9997 for TMZ, confirming a reliable and reproducible absorbance–concentration relationship across the working range. The high R^2^ values validate the suitability of the developed UV spectrophotometric method for the simultaneous estimation of EA and TMZ in the dual-loaded PLGA–zein core–shell nanoparticle formulation. Figure 3 represents the schematic representation of the preparation of FA-TMZ/EA PZ CS NPs.

### 3.2. FT-IR Spectroscopy

**Figure 4:**
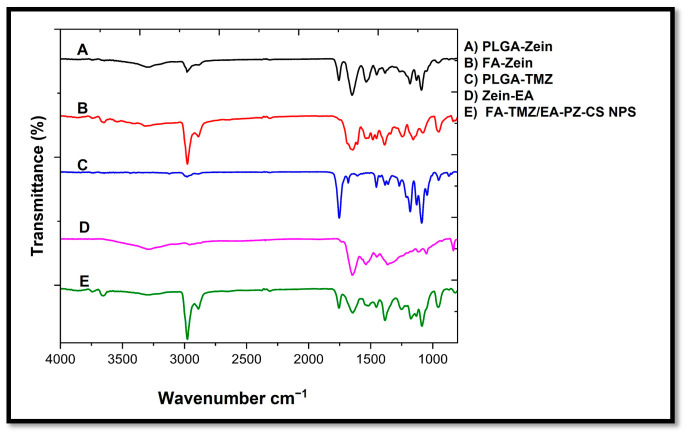
FT-IR spectra of (A) PLGA–Zein Core–shell NPs, (B) Folic acid-conjugated zein NPs, (C) Temozolomide loaded PLGA NPs, (D) Ellagic acid loaded zein NPs, and (E) Folic acid-conjugated dual drug-loaded PLGA–Zein Core–shell NPs.

The FT-IR spectra (Figure S4 and Figure 4) of the individual components (zein, PLGA, Temozolomide, Ellagic acid, and Folic acid), the intermediate formulations (PLGA–zein core–shell NPs, Folic acid-conjugated zein NPs, Temozolomide loaded PLGA NPs, Ellagic acid-loaded zein NPs), and the final dual drug-loaded folic acid-conjugated PLGA–zein core–shell nanoparticles were recorded to evaluate the characteristic functional groups and possible physicochemical interactions. Zein protein showed distinctive amide peaks: Amide I at ~1650 cm^−1^ (C=O stretching), Amide II at ~1530–1545 cm^−1^ (N–H bending coupled with C–N stretching), and Amide III around 1235 cm^−1^ (C–N stretching and N–H bending), confirming its polypeptide nature. Pure PLGA displayed characteristic peaks at ~1755 cm^−1^ corresponding to the strong C=O stretching of ester groups, and bands at ~2940–2990 cm^−1^ and ~1090–1180 cm^−1^ are attributed to the asymmetric/symmetric CH_2_ stretching and C–O–C stretching vibrations, respectively. Temozolomide (TMZ) exhibited its characteristic absorption bands at ~3330–3380 cm^−1^ (NH_2_ and OH stretching), ~1710–1725 cm^−1^ (C=O stretching of imidazotetrazine ring), and weak C–N stretching bands at ~1340–1370 cm^−1^, consistent with previously reported data. EA showed prominent broad O–H stretching bands at ~3380–3450 cm^−1^, strong C=O stretching of lactone groups at ~1720–1730 cm^−1^, and characteristic aromatic C=C stretching at ~1600 cm^−1^ along with peaks around 1050–1150 cm^−1^ corresponding to C–O–C stretching of phenolic groups. FA displayed its characteristic broad N–H/O–H stretching bands at ~3400–3500 cm^−1^, strong C=O stretching of pterin and glutamate moieties at ~1690–1705 cm^−1^, and C–N/C–O bands in the range of ~1200–1300 cm^−1^. In the PLGA–zein core–shell nanoparticles, the prominent ester carbonyl peak of PLGA (~1755 cm^−1^) and the amide bands of zein (~1650 and 1540 cm^−1^) were retained but appeared with reduced intensities and minor shifts (Δ ≈ 2–5 cm^−1^), reflecting effective shell formation without chemical alteration of either polymer. The spectrum of FA-conjugated zein core–shell nanoparticles showed a new, distinct band at ~1540–1560 cm^−1^ (amide linkage) and a decrease in the free carboxylate peak of FA (~1700 cm^−1^), confirming successful EDC/NHS-mediated covalent conjugation of folic acid onto the nanoparticle surface. In the PLGA-TMZ NPs, a significant reduction in the intensity and a slight shift of the C=O stretching peak of PLGA (~1755 cm^−1^ to ~1748 cm^−1^) was observed, accompanied by attenuation of the NH_2_ stretching peak of TMZ (~3330 cm^−1^), suggesting hydrogen-bonding and possible van der Waals interactions between the carbonyl groups of PLGA and the amide moiety of TMZ. The zein–EA interaction was evidenced by a decrease in the intensity and partial broadening of the Amide I band (1650 cm^−1^) and the O-H stretching band of EA (3380 cm^−1^), indicating the formation of hydrogen bonds and hydrophobic interactions between the phenolic hydroxy groups of ellagic acid and the polypeptide backbone of zein. In the final formulation, the characteristic peaks of TMZ (3330 cm^−1^, 1710 cm^−1^) and EA (3380 cm^−1^, 1720 cm^−1^) were significantly suppressed or broadened, and no peaks corresponding to chemical bonds of TMZ and EA were detected. This indicates that both drugs were physically encapsulated within the core–shell structure without covalent modification, while the functional groups of FA and polymers remained intact. The slight shifts and peak broadening in the C=O and N–H regions suggest intermolecular hydrogen bonding and electrostatic interactions, which may contribute to enhanced stability and sustained release.

### 3.3. ^1^H NMR Spectroscopy

The ^1^H NMR spectra (Figure S5) of native zein, folic acid (FA), and FA-conjugated zein nanoparticles (FA-zein NPs) were recorded at 400 MHz to confirm the successful covalent conjugation of FA to the proteinaceous zein backbone via carbodiimide chemistry. The spectrum of zein (Figure S5A) exhibited characteristic resonances corresponding to the proton environments of its amino acid residues. The aliphatic side-chain protons of leucine, isoleucine, valine, and alanine were observed as broad multiplets between δ 0.75–1.10 ppm. The β-CH2 and γ-CH2 protons of glutamine, asparagine, and proline resonated in the δ 1.20–2.30 ppm range. A broad peak around δ 2.70–3.00 ppm was attributed to the methylene protons adjacent to the amide linkages of lysine and arginine residues. The α-protons of the peptide backbone appeared as a broad resonance at δ 4.00–4.40 ppm, while a weak and broad signal between δ 7.00–8.20 ppm corresponded to the backbone amide NH protons, which were broadened due to hydrogen bonding and the semi-crystalline nature of zein. The spectrum of FA (Figure S5B) displayed the diagnostic signals of the pteridine and p-aminobenzoate moieties. The singlet of the pteridine H-2 proton appeared at δ 8.55 ppm, and the singlet corresponding to H-8 was noted at δ 8.20 ppm. The aromatic protons of the p-aminobenzoic acid ring resonated as doublets at δ 7.65 ppm and δ 6.65 ppm, corresponding to the ortho- and meta-positions relative to the amino group. The characteristic –CH2– protons of the glutamate side chain were observed as multiplets at δ 2.10–2.45 ppm (β-CH2) and δ 4.25 ppm (α-CH). A broad resonance between δ 11.0–12.0 ppm corresponded to the –NH and –COOH protons, reflecting the hydrogen-bonding interactions in FA. FA-Conjugated zein Nanoparticles spectrum (Figure S5C) retained most of the characteristic resonances of zein but exhibited distinct additional peaks corresponding to FA, confirming successful conjugation. New aromatic signals appeared at δ 8.50–8.10 ppm and δ 7.60–6.60 ppm, matching the chemical shifts of the FA pteridine and p-aminobenzoate protons, which were absent in native zein. Moreover, the resonance of the –CH2– group adjacent to the carboxyl group of the FA glutamate shifted slightly downfield to δ 2.40 ppm, indicating a change in the local chemical environment upon conjugation. The amide NH protons broadened and shifted slightly from δ 7.00–8.20 ppm to δ 7.10–8.30 ppm, consistent with new amide bond formation between the carboxyl group of FA and the amino groups of lysine residues in zein. The reduced intensity of the free-COOH proton signal of FA at δ ~11.5 ppm further supported its covalent attachment. Figure S5D illustrates the chemical mechanism of covalent bond formation in between FA and zein by using EDC/NHS. These spectral features, together with the disappearance of sharp free FA peaks, confirmed the formation of stable FA–zein conjugates.

### 3.4. Differential Scanning Calorimetry

DSC thermograms (Figure S6) of the individual components, physical excipients, and the final nanoparticle formulation were obtained to assess thermal transitions, crystallinity, and potential molecular-level interactions among the formulation constituents. Zein exhibited a broad endothermic transition with an onset at 32.8 °C and a peak at 85.27 °C, followed by completion at 124.89 °C, corresponding to protein structural relaxation and partial denaturation, with an associated enthalpy of 208.29 J/g. PLGA displayed two distinct endothermic events, first at 56.13 °C (onset: 24.92 °C, endset: 64.57 °C, ΔH: 39.09 J/g), indicative of glass transition-related thermal relaxation, and a second broader melting-associated endotherm at 116.76 °C (onset: 108.35 °C, end set: 121.40 °C, ΔH: 7.54 J/g) consistent with semi-crystalline polymer domains. Temozolomide displayed a pronounced exothermic peak at 217.70 °C (onset: 202.32 °C; endset: 212.71 °C with a high associated enthalpy of 414.60 J/g, consistent with reported thermal decomposition rather than classical melting behaviour. EA exhibited a sharp endothermic peak at 144.35 °C (onset: 21.30 °C, endset: 153.84 °C, ΔH: 181.63 J/g), confirming its crystalline nature. FA showed two endothermic transitions: a minor event at 168.64 °C (ΔH: 18.44 J/g) and a major melting-associated event at 198.43 °C (onset: 186.69 °C, endset: 228.78 °C, ΔH: 225.89 J/g) indicative of polymorphic crystalline domains. In contrast, the FA-TMZ/EA-PZ-CS nanoparticles demonstrated a single broad endothermic transition at 304.34 °C (onset: 300.94 °C; endset: 311.24 °C) with significantly reduced enthalpy (108.93 J/g) compared to the pure individual constituents. The disappearance of the characteristic melting peaks of temozolomide, EA, and FA, along with the shift in PLGA and zein transitions, indicates loss of crystalline structure and successful molecular embedding of the actives within the polymer–protein matrix. The shift to a higher transition temperature suggests enhanced thermal stability of the final nanoformulation.

### 3.5. X-Ray Diffraction Studies

The XRD diffractograms of zein, PLGA, TMZ, EA, FA, zein–PLGA core–shell nanoparticles, and FA-conjugated dual drug-loaded zein–PLGA nanoparticles are presented in Figure S7. Zein displayed a broad diffuse peak centred around 2θ ≈ 19.6°, characteristic of its amorphous nature. PLGA showed a similar broad hump at 2θ ≈ 16.8°, indicating a non-crystalline polymeric matrix. Pure TMZ exhibited distinct sharp diffraction peaks at 2θ values of 9.3°, 13.8°, 17.2°, and 28.1°, confirming its highly crystalline structure. EA also demonstrated multiple intense peaks at 2θ ≈ 15.7°, 23.6°, 26.2°, and 28.9°, corresponding to the crystalline lattice arrangement of the material. Folic acid displayed characteristic reflections at 2θ ≈ 12.1°, 19.3°, and 25.8°, confirming its semi-crystalline nature. In contrast, the zein–PLGA core–shell nanoparticles exhibited a broad halo pattern without prominent diffraction peaks, revealing a complete loss of crystallinity of the encapsulated drugs. Similarly, the FA-TMZ/EA-ZP-CS nanoparticles showed a diffused pattern with a broad hump between 15° and 25°, indicating that TMZ and EA were molecularly dispersed within the amorphous polymeric matrix. No distinct peaks corresponding to the pure drugs or folic acid were observed, suggesting successful encapsulation and molecular-level entrapment in the nanosystem.

### 3.6. Drug Release and Release Kinetics

The in vitro release profiles (Figure S8) of TMZ and EA were evaluated at physiological (pH 7.4) and mildly acidic (pH 5.5) conditions, simulating the tumour microenvironment and endosomal/lysosomal compartments. Both drugs exhibited a biphasic release pattern, characterised by an initial burst phase within the first 8 h, followed by sustained, controlled release up to 120 h. At pH 7.4, TMZ release reached 67.86% after 120 h, while EA release reached 81.55%, indicating effective encapsulation within the PLGA–zein matrix. Under acidic conditions (pH 5.5), a significantly faster release was observed, with TMZ and EA reaching 85.89% and 91.39%, respectively. The enhanced drug diffusion at pH 5.5 is attributed to accelerated PLGA polymer swelling and hydrolytic degradation, as well as partial zein protonation, which facilitate easier drug diffusion. The dual-drug system thus demonstrated pH-responsive behaviour favourable for selective drug release in the tumour milieu. Drug release kinetics (Table S2) of the TMZ/EA-PZ-CS nanosystem were evaluated at physiological (pH 7.4) and tumour-mimicking acidic conditions (pH 5.5). Across all conditions, the release profiles showed the highest correlation with the Korsmeyer–Peppas model, with R^2^ values ranging from 0.9926 to 0.9982, indicating a predominant anomalous (non-Fickian) diffusion mechanism. At pH 7.4, TMZ exhibited R^2^ = 0.9926, while EA showed R^2^ = 0.9941. Under acidic conditions, an even stronger fit was observed (TMZ R^2^ = 0.9974, EA R^2^ = 0.9982), confirming enhanced diffusion-controlled release. The Higuchi model displayed the second-highest correlation (TMZ: 0.9837 at pH 7.4; 0.9696 at pH 5.5; EA: 0.9668 at pH 7.4; 0.9272 at pH 5.5), supporting a sustained, matrix-driven release. First-order kinetics (R^2^ = 0.9086–0.9614) indicated concentration-dependent release, whereas zero-order kinetics showed a weaker correlation (R^2^ = 0.7483–0.9023), indicating that drug release was not constant over time. The sustained, pH-responsive release profile validates the potential of the folate-guided PLGA–zein nanosystem for controlled and site-specific co-delivery of chemotherapeutic and PARP-inhibitor agents in glioblastoma therapy.

### 3.7. Blood Compatibility

The in vitro hemolysis assay using human erythrocytes demonstrated excellent blood compatibility of the nanoformulations. Figure S9 shows the hemolysis results of FA-TMZ/EA-PZ-CS NPs. The percentage hemolysis values of erythrocytes treated with a range of sample concentrations (10–1000 μg/mL of drug-loaded NPs) were comparable to those of the negative control, saline, and all these values were less than 5%, as per ASTM standards. Thus, the hemocompatibility results for FA-TMZ/EA-PZ-CS NPs suggest the potential of these NPs for intravenous administration.

### 3.8. Biocompatibility Assessment of FA-TMZ/EA-PZ-CS NPs on L929 Cells Using MTT Assay

The cytocompatibility of the FA-TMZ/EA-PZ-CS NPs was evaluated using the MTT assay on L929 fibroblast cells over a concentration range of 10–1000 µg/mL. As shown in Figure S10, the nanosystem exhibited excellent cell viability, with more than 80% of viable cells observed across all tested concentrations. Even at the maximum concentration of 1000 µg/mL, cell viability remained above 80%, indicating negligible cytotoxicity, which is the threshold defined by ISO 10993-5 standards for biomedical materials. These findings confirm that the folic acid-conjugated PLGA–zein nanosystem is highly cytocompatible and safe towards normal fibroblast cells within the evaluated dose range.

### 3.9. Synergistic Evaluation of Temozolomide and Ellagic Acid on LN229 Glioblastoma Cells

**Figure 5:**
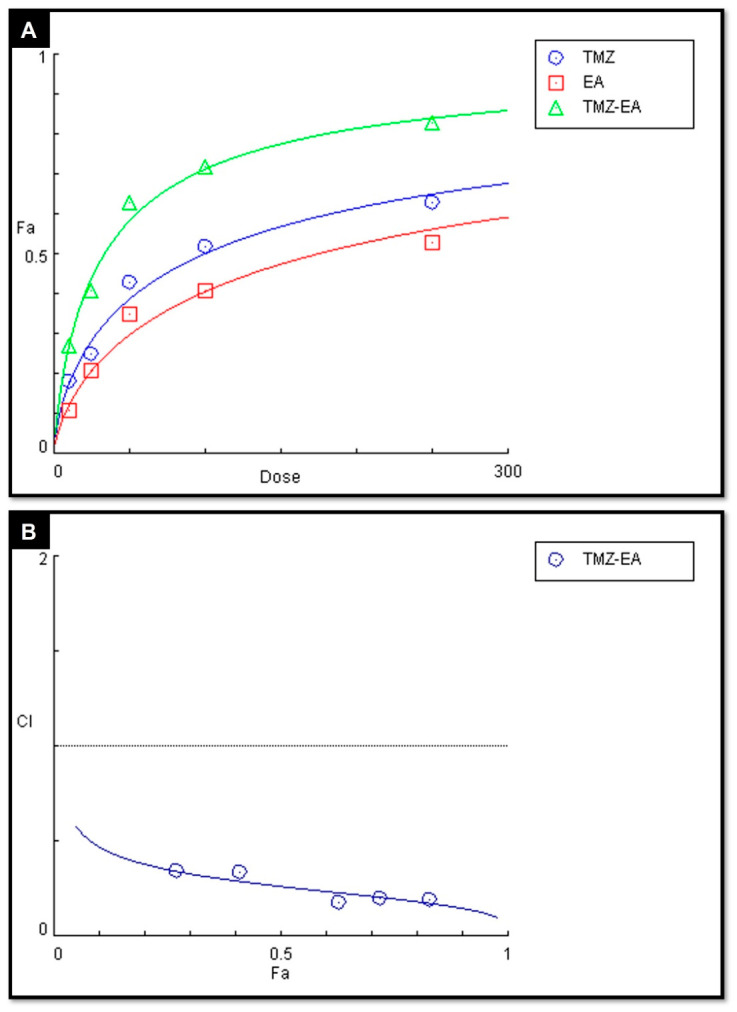
The synergistic effect of TMZ and EA on the viability of LN229 GBM cells. (**A**) Dose–effect curve. (**B**) Combination index plot. Data are presented as mean ± SD (n = 3 independent experiments, each performed in triplicate wells).

The cytotoxic effects of TMZ, EA, and their fixed-dose combination (TMZ + EA in a 1:1 ratio) were quantified using the MTT assay and analysed using CompuSyn software following the Chou–Talalay method. The dose effect plot (Figure 5A) explains the cytotoxic effect of each treatment group on LN229 cells at various doses. TMZ alone led to a progressive reduction in cell viability, with an IC50 of 95.613 μg/mL, indicating moderate inherent chemosensitivity. EA exhibited a weaker cytotoxic profile with an IC_50_ of 172.584 μg/mL, indicating that higher concentrations were required to achieve comparable inhibitory effects. Meanwhile, co-administration of TMZ and EA significantly enhanced cytotoxicity, reducing the IC_50_ to 32.475 μg/mL. This highlights a potential impact of EA, sensitising GBM cells to TMZ and thereby lowering the effective dose required for cytotoxicity. The fraction affected (Fa) versus combination index (CI) study (Figure 5B) revealed CI values consistently less than one at all concentration levels, demonstrating a strong synergistic interaction. Early synergy was observed at lower dosages (CI = 0.3455 and 0.3399 at Fa = 0.27 and 0.41, respectively), and it increased significantly with increasing effect levels. Medium- and high-level dose combinations produced strong synergy (CI = 0.180–0.196 across FA = 0.63–0.83), demonstrating a dose-amplified cooperative effect rather than a simple additive response. The lower CI trend with increasing Fa further supported the synergy profile, indicating that as tumour cell inhibition increases, the therapeutic interaction becomes more effective.

The isobologram (Figure S11A) provides a graphical representation of the pharmacodynamic interaction between TMZ and EA at equivalent effect levels. The IC_50_ values of the individual drugs are plotted on the X and Y axis, and a straight line connecting these points indicates the theoretical additive line of interaction. Drug combinations that fall on the line represent additive effects. Points above the line indicate antagonism, and points below the line show synergy. In this study, all TMZ-EA combination dose points were located below the line of additivity, indicating a strong synergistic interaction rather than an additive effect. The greater downward deviation observed at higher fractional inhibition levels (Fa ≥ 0.50) represents enhanced synergistic effects, which is consistent with CI and DRI results. The combination points to the lower left quadrant of the plot, showing that the treatment can achieve the same biological effects using lower drug doses, which highlights its dose-sparing benefit. This visualisation, therefore, confirms that EA enhances TMZ potency beyond expected additive behaviour and strengthens the inference that mechanistic complementarity underlies the observed cytotoxic amplification. Dose-reduction index (DRI) analysis further validated the therapeutic advantage of the combination, demonstrating significant dose-sparing effects for both agents.

TMZ exhibited a 4.48-fold reduction in the required dose at Fa = 0.27, increasing to 8.44-fold at Fa = 0.83. Similarly, EA demonstrated notable dose efficiency, with DRI values ranging from 8.17 at lower inhibition to 15.04 at mid-level and stabilizing above 13 at higher Fa values. The increasing DRI trend, accompanied by a rising biological effect, indicates that combined treatment not only enhances cytotoxicity but also substantially reduces the drug burden required to achieve therapeutic efficacy. Figure S11B represents a median-effect plot generated with CompuSyn, which displayed a strong linear relationship for TMZ, EA, and the TMZ–EA combination, confirming that the dose–response behaviour of all treatments conformed to the mass-action law. The regression coefficients (r = 0.98655 for TMZ, 0.98488 for EA, and 0.99262 for the combination) demonstrated excellent linearity, validating the robustness of dataset modelling and the reliability of subsequent synergy calculations. The shift of the combination curve toward the left relative to the single-drug plots indicates enhanced potency, while the steeper slope reflects a more cooperative inhibitory pattern. These characteristics further support that the co-administration of TMZ and EA produces a significantly stronger cytotoxic response than expected from the individual agents, aligning with the reduced Dm and the synergy profile demonstrated in CI and DRI analyses.

**Figure 6:**
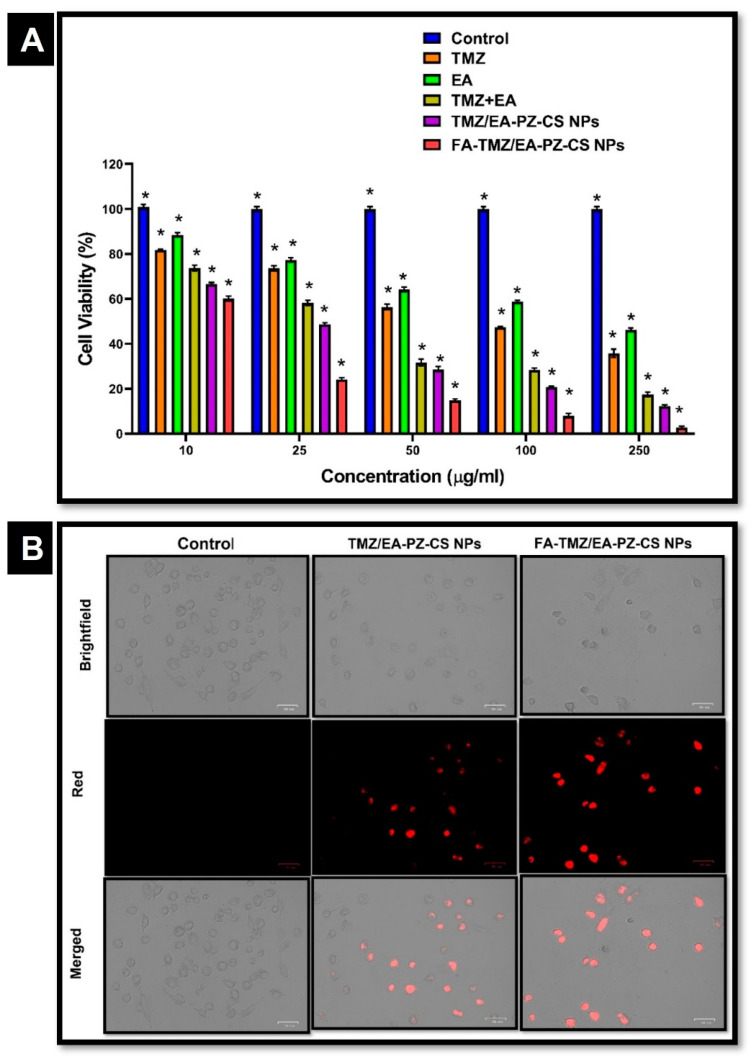
(**A**) Cytotoxicity evaluation of TMZ, EA, TMZ + EA, TMZ/EA-PZ-CS NPs, FA-TMZ/EA-PZ-CS NPs on LN229 cells. Data are presented as mean ± SD (*n* = 3 independent experiments, each performed in triplicate wells). Statistical analysis performed by one-way ANOVA followed by Tukey’s post hoc test; * *p* < 0001. (**B**) Fluorescence microscopy images of LN229 glioma cells treated with control, rhodamine 123–labelled non-conjugated TMZ/EA-PZ-CS NPs, and rhodamine 123–labelled FA-TMZ/EA-PZ-CS NPs. The scale bar represents 100 μm.

Figure 6A illustrates the data of cytotoxicity assessment of TMZ, EA, and the TMZ–EA combination in LN229 cells using the MTT assay. The untreated control group exhibited consistent cell viability, ranging from 95% to 100%, confirming normal cell line proliferation and assay stability. Both TMZ and EA treatments demonstrated a concentration-dependent decline in cell viability. TMZ monotherapy reduced viability from approximately 81–83% at low doses to 36–38% at the highest doses, indicating a progressive cytotoxic response. EA exhibited a similar decreasing trend, though initial reductions were less pronounced (88–90% viability at the lowest dose), with viability decreasing to 46% at the highest concentration. In contrast, the TMZ + EA combination produced a markedly greater inhibitory effect at all corresponding exposure levels. Cell viability decreased to approximately 72% at the lowest combination dose and to 56% at intermediate doses. The most substantial response was observed at the highest dose combination, where viability decreased to 16–19%, representing a significantly stronger anticancer effect relative to TMZ or EA alone.

### 3.10. Cytotoxicity Evaluation of Nanoformulations

The cytotoxic potential of TMZ/EA-PZ-CS NPs and FA-TMZ/EA-PZ-CS NPs was evaluated using the MTT assay across a concentration range of 10–250 µg/mL, represented in Figure 6A. Both formulations demonstrated concentration-dependent reductions in LN229 cell viability; however, the FA-conjugated formulation exhibited significantly greater cytotoxicity than the non-conjugated counterpart. For the non-conjugated TMZ/EA-PZ-CS NPs, the percentage of viable cells decreased progressively from 66.7 ± 0.76% at the lowest dose (10 µg/mL) to 12.2 ± 0.65% at the highest concentration tested (250 µg/mL). The decrease in viability at intermediate concentrations was consistent and statistically significant, with values of 48.7 ± 0.60% at 25 µg/mL, 28.8 ± 1.07% at 50 µg/mL, and 20.7 ± 0.38% at 100 µg/mL, demonstrating effective dose-dependent antiproliferative action attributable to the synergistic mechanism of TMZ and EA. In contrast, the FA-TMZ/EA-PZ-CS nanoparticles exhibited substantially enhanced cytotoxicity at all tested concentrations. At 10 µg/mL concentration, cell viability decreased to 60.5 ± 1.14%, and a further drop to 24.2 ± 0.80% was recorded at 25 µg/mL. A pronounced improvement in sensitivity was observed at concentrations of 50 µg/mL and above, where viability reduced to 14.6 ± 0.50% at 50 µg/mL and 8.09 ± 0.98% at 100 µg/mL. Notably, at the highest dose (250 µg/mL), only 2.77 ± 0.52% viable cells remained, representing a fourfold improvement in cytotoxic potency compared with the non-conjugated formulation at the same concentration. IC_50_ values were calculated from nonlinear regression of the dose–response curves. The non-conjugated TMZ/EA-PZ-CS NPs exhibited an IC_50_ value of 21.73 µg/mL, indicating effective cytotoxic activity within the tested dose range. In contrast, the FA-TMZ/EA-PZ-CS NPs demonstrated a substantially lower IC_50_ value of 12.29 µg/mL, confirming markedly enhanced potency and improved cellular response attributable to receptor-mediated targeting. The nearly 1.8-fold reduction in IC_50_ underscores the role of folate surface modification in facilitating increased intracellular accumulation and enhancing therapeutic efficacy against glioblastoma cells.

### 3.11. Cellular Uptake Studies of Nanoformulations

Cellular uptake of rhodamine-labelled TMZ/EA-PZ-CS NPs and FA-TMZ/EA-PZ-CS NPs was visualised using fluorescence microscopy (Figure 6B) to assess intracellular internalization in LN229 glioblastoma cells. The control cells, treated only with culture medium, exhibited negligible fluorescence, confirming minimal background signal. Cells treated with non-conjugated nanoparticles showed observable intracellular fluorescence, indicating successful nanoparticle uptake. Cells treated with FA-TMZ/EA-PZ-CS NPs appeared to exhibit stronger intracellular fluorescence compared with the non-conjugated formulation, qualitatively suggesting enhanced cellular internalization. In both treated groups, fluorescence was distributed predominantly within the cytoplasmic region, supporting intracellular localization of the nanosystems.

### 3.12. Trypan Blue Dye Exclusion Assay

A Trypan Blue dye exclusion assay (Figure S12) was performed to evaluate the effect of free and folate-targeted dual drug-loaded nanoparticles on LN229 cell viability following treatment. The untreated control group demonstrated high viable cell counts with an average of 79,000 ± 200 cells, indicating normal cellular proliferation in the absence of treatment. Treatment with non-targeted TMZ/EA-PZ-CS nanoparticles resulted in a marked reduction in viable cells, with an average viable cell count of 42,733 ± 208 cells, reflecting a substantial loss in viability compared to the control, while cells treated with FA-TMZ/EA-PZ-CS nanoparticles exhibited the greatest reduction in cell viability, with an average viable cell count of 30,766 ± 251 cells, indicating enhanced cytotoxicity relative to the non-targeted formulation. One-way ANOVA followed by Tukey’s post hoc test was performed to compare cell viability between treatment groups. A statistically significant reduction in cell viability was observed for both TMZ/EA-PZ-CS nanoparticles and FA-TMZ/EA-PZ-CS nanoparticles when compared with the untreated control group (*p* < 0.0001). Furthermore, FA-TMZ/EA-PZ-CS nanoparticles displayed significantly greater cytotoxicity compared to the non-targeted TMZ/EA-PZ-CS nanoparticles (*p* < 0.0001). These findings further substantiate the superior therapeutic efficacy of the FA-targeted nanocarrier system.

### 3.13. Clonogenic Assay

**Figure 7:**
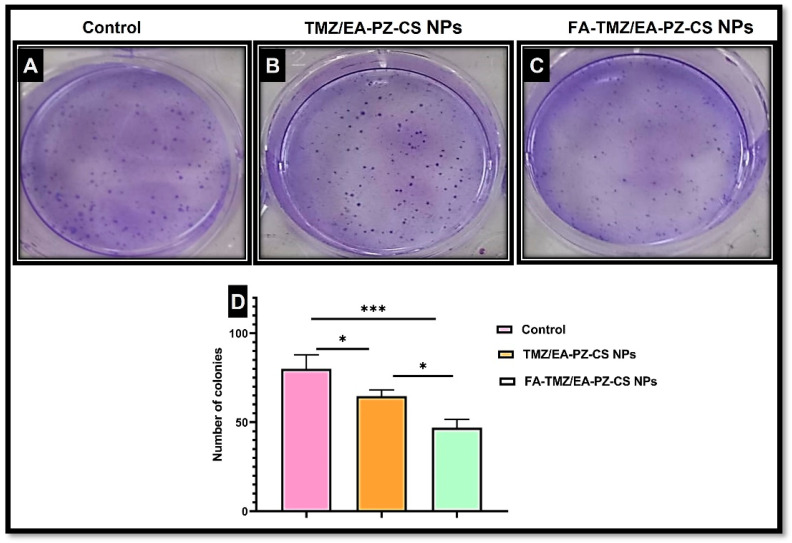
Quantification of clonogenic survival of LN229 cells after treatment with (**A**) control, (**B**) TMZ/EA-PZ-CS NPs, and (**C**) FA-TMZ/EA-PZ-CS NPs. Quantitative bar graph (**D**) representing clonogenic survival of LN229 cells following treatment with control, non-targeted TMZ/EA-PZ-CS NPs, and folic acid-conjugated FA-TMZ/EA-PZ-CS NPs. Results are expressed as mean ± SD (n = 3). Statistical analysis was performed using one-way ANOVA followed by Tukey’s post-hoc test (* *p* < 0.05, *** *p* < 0.001).

The clonogenic assay was performed to evaluate the long-term proliferative capacity of LN229 cells following treatment with non-targeted TMZ/EA-PZ-CS nanoparticles and targeted FA-TMZ/EA-PZ-CS nanoparticles. The representative images of colony growth for each group are shown in Figure 7A–C. Quantitative bar graph (Figure 7D) representing clonogenic survival of LN229 cells following treatment with control, non-targeted TMZ/EA-PZ-CS nanoparticles, and folic acid-conjugated FA-TMZ/EA-PZ-CS nanoparticles. The untreated control group exhibited dense and distinct colonies, with an average colony count of 80 ± 7.8, indicating a high inherent clonogenic potential. In contrast, treatment with non-targeted TMZ/EA-PZ-CS nanoparticles resulted in a significant reduction in colony formation, yielding an average of 64.6 ± 3.7 colonies, indicating a suppression of cellular self-renewal capacity. Notably, the folic acid-modified system (FA-TMZ/EA-PZ-CS NPs) demonstrated the strongest inhibitory effect on cell clonogenicity, with a marked decline in colony count to 47 ± 4.5, representing approximately 41.2% inhibition relative to control, compared to 19.3% inhibition observed with non-conjugated formulations.

### 3.14. Hoechst Staining

**Figure 8:**
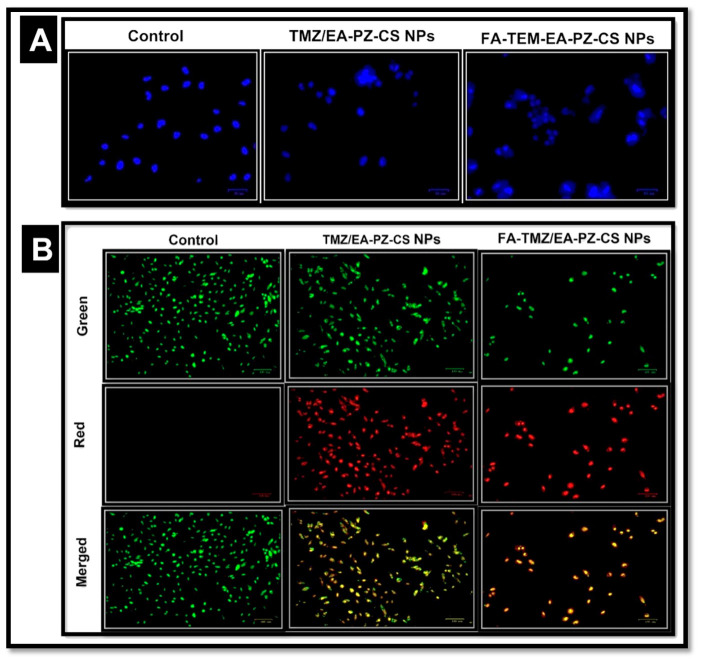
(**A**) Hoechst 33342 staining demonstrating nuclear morphological changes in LN229 cells after treatment with dual drug-loaded nanosystems. (**B**) AO/EB dual staining images of LN229 cells treated with Control, Non-conjugated dual drug-loaded zein–PLGA nanoparticles, and FA-conjugated dual drug-loaded zein–PLGA nanoparticles. The scale bar represents 100 µm.

Hoechst 33342 staining (Figure 8A) was performed to qualitatively assess nuclear morphology and apoptotic changes induced by various nanoparticle formulations in LN229 glioma cells. The untreated control cells exhibited round, intact, and uniformly stained nuclei, indicating normal nuclear morphology and the absence of apoptotic features. Cells treated with non-conjugated TMZ/EA-PZ-CS NPs displayed a moderate increase in fluorescence intensity, along with evidence of nuclear condensation and a few fragmented nuclei, suggesting the onset of apoptosis. In contrast, cells exposed to FA-TMZ/EA-PZ-CS NPs exhibited pronounced nuclear fragmentation, chromatin condensation, and bright blue fluorescence, characteristic hallmarks of nuclear damage. The intensity and abundance of apoptotic nuclei were significantly higher in the folic acid-conjugated group compared with both the non-conjugated nanoparticle-treated and control groups, confirming enhanced apoptotic induction by the targeted system. These morphological alterations were consistent with the results of the MTT and clonogenic assays, validating the superior therapeutic efficacy of the folate-targeted dual-drug nanosystem in LN229 glioblastoma cells.

### 3.15. AO/EB Staining for Apoptosis Detection

The apoptotic effects of the developed formulations on LN229 glioblastoma cells were evaluated using Acridine Orange/Ethidium Bromide (AO/EB) dual staining (Figure 8B). Distinct fluorescence patterns were observed among the control, non-conjugated, and folic acid-conjugated nanosystem-treated groups, reflecting the stage of apoptosis and membrane integrity. In the control group, cells exhibited uniform green fluorescence with intact nuclei, indicating normal morphology and viability. In contrast, cells treated with the non-conjugated dual drug-loaded PLGA-Zein core-shell nanoparticles exhibited a mixture of green and yellow-orange fluorescence, indicating the onset of early apoptosis characterised by chromatin condensation and partial membrane permeabilisation. A marked difference was observed in cells treated with FA-conjugated dual drug-loaded PLGA-Zein core-shell nanoparticles, which exhibited intense orange to reddish-orange fluorescence. These cells exhibited classical apoptotic features, including nuclear fragmentation, chromatin condensation, and membrane blebbing, indicative of late apoptosis and secondary necrosis.

### 3.16. Quantitative Protein-Level Validation of PARP-1 Suppression

**Figure 9:**
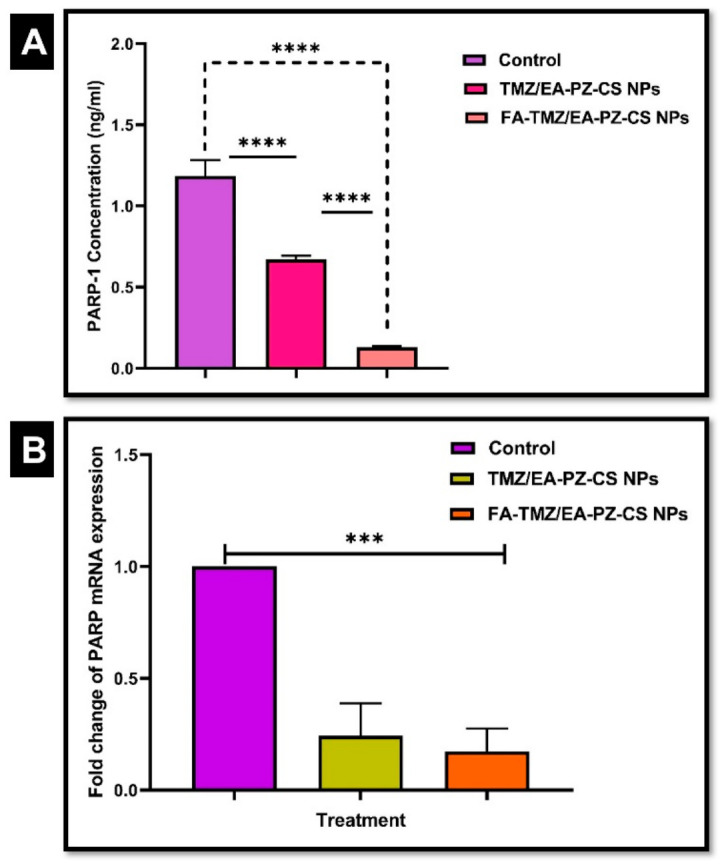
(**A**) Assessment of PARP-1 concentration measurement by ELISA kit. Data represent mean ± SD (*n* = 3). Statistical analysis performed by one-way ANOVA followed by Tukey’s post hoc test; **** *p* < 0.0001. (**B**) Changes in the gene expression of PARP post-treatment with TMZ/EA-PZ-CS NPs and FA-TMZ/EA-PZ-CS NPs. Statistical analysis performed by one-way ANOVA followed by Tukey’s post hoc test. *** *p* < 0.001.

PARP-1 expression levels were quantified using an ELISA kit to provide quantitative protein-level validation of the ability of the formulated nanosystems to inhibit poly (ADP-ribose) polymerase activity, a key enzyme involved in DNA repair mechanisms that contributes to TMZ resistance in glioblastoma. The protein concentrations were expressed as ng/mL. The absorbance was measured at 450 nm, where a lower absorbance indicates stronger PARP inhibition. As shown in Figure 9A, untreated control cells exhibited the highest endogenous PARP-1 levels (1.18 ± 0.10 ng/mL). Treatment with non-targeted TMZ/EA-PZ-CS nanoparticles resulted in a significant reduction of PARP-1 expression (0.67 ± 0.02 ng/mL), corresponding to approximately 43–45% suppression relative to the control group. Significantly, cells treated with FA-TMZ/EA-PZ-CS NPs demonstrated a markedly greater inhibitory effect, with PARP-1 levels reduced to 0.13 ± 0.01 ng/mL, representing approximately 88–90% reduction compared to untreated cells. Statistical analysis confirmed that both nanoparticle formulations significantly reduced PARP-1 levels compared to the control (*p* < 0.001). Furthermore, FA-TMZ/EA-PZ-CS NPs exhibited statistically superior inhibition compared to non-targeted nanoparticles (*p* < 0.01), indicating improved therapeutic efficacy attributable to folate-mediated targeting and enhanced intracellular delivery.

### 3.17. Gene Expression Analysis Using qRT-PCR

Quantitative real-time PCR analysis was performed to evaluate the impact of various nanoparticle formulations on PARP mRNA expression. As shown in Figure 9B, the untreated control cells exhibited basal expression levels normalized to 1.0. Treatment of LN229 cells with non-targeted TMZ/EA-PZ-CS NPs resulted in a significant downregulation of PARP expression levels, compared to the control (*** *p* < 0.001). A further downregulation in PARP expression was observed in cells treated with FA-EA-PZ-CS NPs, suggesting that functionalization with FA improves the anticancer efficacy of the formulation, mediated through enhanced cellular uptake and targeted delivery.

### 3.18. Comet Assay

**Figure 10:**
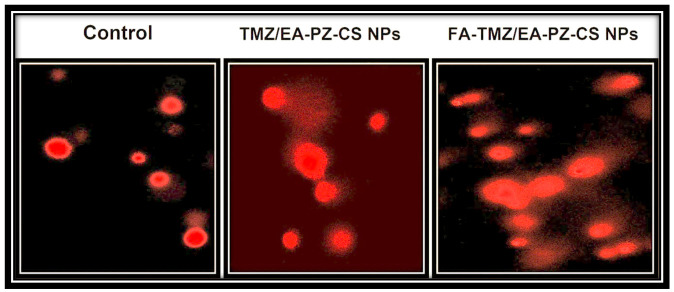
Comet assay demonstrating DNA fragmentation in LN229 cells following treatment with control, TMZ/EA-PZ-CS nanoparticles, and FA-TMZ/EA-PZ-CS nanoparticles.

The alkaline comet assay was employed to quantify DNA strand breaks in LN229 glioblastoma cells following 24 h treatment with TMZ/EA-PZ-CS NPs and FA-TMZ/EA-PZ-CS NPs, with untreated cells serving as the negative control. Microscopic examination (Figure 10) revealed almost intact, round nuclei with negligible or no comet tails in control cells, indicating minimal baseline DNA damage.

**Figure 11:**
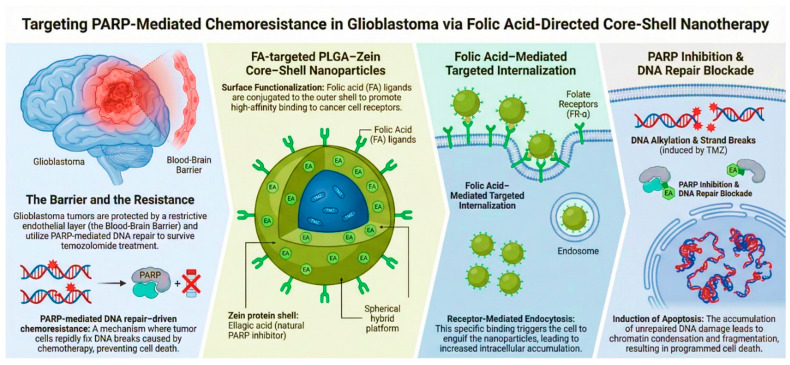
Represents the cellular mechanism of dual drug-loaded FA-targeted PLGA–zein Core–shell nanoparticles.

Cells exposed to non-conjugated TMZ/EA-PZ-CS NPs caused a pronounced increase in comet frequency and tail intensity, demonstrating enhanced DNA damage attributable to the nanoparticle-facilitated co-delivery and sustained intracellular release of both agents. In contrast, FA-TMZ/EA-CS NPs elicited the most substantial genotoxic response, with a higher proportion of cells displaying long and dense comet tails compared with all other groups, signifying pronounced DNA strand scission. Figure 11 represents the molecular mechanism of folic acid targeted dual drug loaded PLGA-Zein core shell nanoparticles after cellular internalisation. Further PARP inhibition leads to apoptosis or necrosis.

### 3.19. Cell Migration (Scratch Assay)

**Figure 12:**
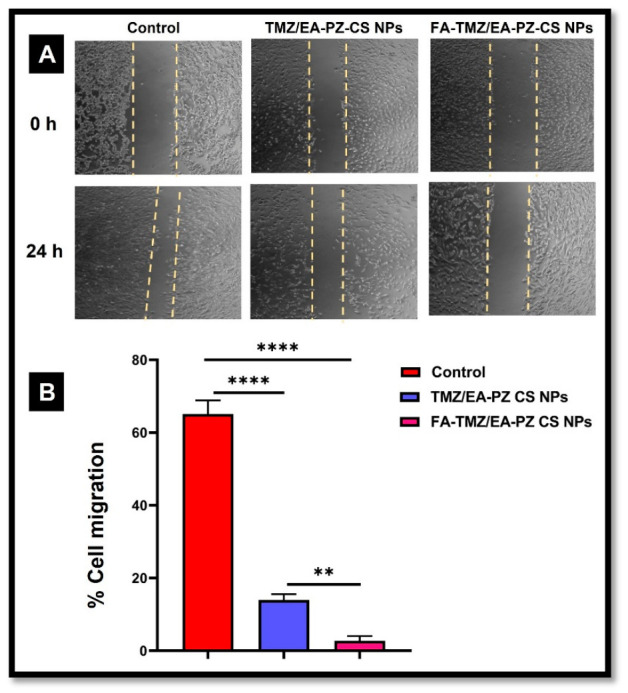
(**A**) Cell migration analysis of LN229 cells treated with control, TMZ/EA-PZ CS NPs, FA–TMZ/EA-PZ CS NPs. Phase-contrast microscopic images showing cell migration at 0 h and 24 h for (**A**) control cells, non-conjugated TMZ/EA PZ–CS NPs treated cells, and targeted FA–TMZ/EA-PZ CS NPs treated cells. (**B**) Quantitative analysis of the percentage of cell migration after 24 h demonstrates a significant reduction in cell migration in the FA–TMZ/EA PZ–CS NP-treated group compared to control and non-conjugated nanoparticles (** *p* < 0.01, **** *p* < 0.0001 one-way ANOVA). Data are expressed as mean ± SD (n = 3).

The migratory behaviour (Figure 12A) of LN229 glioma cells was evaluated using the scratch wound assay at 0 h and 24 h following treatment with control (untreated cells), non-conjugated dual drug-loaded zein–PLGA nanoparticles (TMZ/EA PZ-CS NPs), and folic acid-conjugated dual drug-loaded zein–PLGA nanoparticles (FA-TMZ/EA PZ-CS NPs). Quantitative analysis (Figure 12B) of wound closure revealed a substantial reduction in cell migration following treatment with nanoformulations compared to the untreated control. The control group exhibited a mean migration rate of 65.09% ± 3.85, indicating rapid wound closure. In contrast, cells treated with non-targeted TMZ/EA-PZ-CS NPs demonstrated significantly reduced migration (13.94% ± 1.64, *p* < 0.001), confirming the inhibitory effect of the dual-drug nanoformulation on cellular motility. Notably, the folate-guided targeted nanoformulation (FA-TMZ/EA-PZ-CS NPs) exhibited the strongest migration inhibition, with a mean migration rate of only 2.74% ± 1.24 (*p* < 0.001 vs. control and non-targeted NPs). Statistical analysis using one-way ANOVA followed by Tukey’s post hoc test confirmed significant differences among treatment groups (*p* < 0.05). These findings demonstrate that folic acid conjugation further enhances the therapeutic efficacy of the nanoformulation by improving targeting and reducing cellular migration.

## 4. Discussion

The PLGA–zein core–shell nanoparticles were successfully fabricated using a two-step nanoprecipitation approach. In the first phase, the organic solution containing PLGA was added dropwise to an aqueous phase under constant stirring, resulting in spontaneous nucleation and the formation of nanosized PLGA cores via solvent diffusion and subsequent polymer precipitation [15]. The formation of colloidally stable primary PLGA nanoparticles confirmed the suitability of the optimised acetone:water ratio and stirring conditions for efficient nanoprecipitation. Subsequently, zein was introduced in ethanol and added to the preformed PLGA cores, followed by controlled acidification to induce zein precipitation around the PLGA particles, forming a uniform secondary protein shell [16]. The interaction between the hydrophobic domains of zein and the polymeric core structure facilitated stable assembly and a core–shell architecture. The resulting plain PLGA–zein nanoparticles exhibited a well-defined spherical morphology with smooth boundaries, affirming successful surface coating and structural integrity. In the core–shell nano system, TMZ and the hydrophobic natural PARP inhibitor EA were incorporated during the respective formation stages to maximize compartmentalization and prevent cross-diffusion between layers. TMZ was encapsulated within the PLGA matrix during nanoprecipitation, leveraging polymer–drug interactions and diffusion-driven entrapment, while the second drug was loaded into the zein layer during protein assembly through hydrophobic and hydrogen bonding interactions. The sequential encapsulation strategy resulted in efficient spatial localization of the two therapeutics, minimizing burst release and enabling sustained delivery [17]. The dual drug-loaded nanoparticles displayed no observable aggregation or phase separation, indicating that drug incorporation did not adversely affect shell formation or colloidal stability [18,19]. A modest increase in particle size was observed upon dual-drug incorporation, reflecting effective drug integration within the PLGA core and the zein shell. The core–shell architecture further enhanced structural robustness, highlighting the suitability of this nanoplatform for sustained, synergistic drug delivery in glioblastoma treatment. TEM images correlated strongly with the DLS results, providing visual confirmation of the spherical morphology, core–shell configuration, and uniform distribution of nanoparticles. The consistent round geometry and intact shell surrounding the PLGA core demonstrate successful precipitation-driven assembly, confirming that drug incorporation did not disrupt structural formation [20].

FT-IR spectra confirmed successful formation of the PLGA–zein core–shell system, as the characteristic PLGA carbonyl and zein amide peaks were retained with only minor shifts, indicating non-covalent polymer interactions. The appearance of a new amide band and loss of the free-COOH peak verified covalent folic-acid conjugation through EDC/NHS chemistry. Broadening and narrowing of TMZ and EA characteristic peaks in the final nanoparticles, without the emergence of new absorption bands, confirmed the physical encapsulation of both drugs. The slight shifts in the C=O and N–H regions reflect intermolecular hydrogen bonding, contributing to enhanced nanosystem stability and controlled release [21,22]. The covalent conjugation of folic acid (FA) to zein via EDC/NHS chemistry was conclusively verified by ^1^H-NMR spectroscopy. Activation of the γ-carboxyl group of FA enabled selective amide bond formation with ε-amino groups of lysine residues in zein. The appearance of FA-specific aromatic proton signals in the FA–zein spectrum, absent in native zein, together with downfield shifting and broadening of amide-associated resonances, provided direct evidence of covalent attachment. The marked attenuation of the FA–COOH proton signal (δ 11–12 ppm) further confirmed its consumption during amidation. Subtle deshielding of FA’s glutamate α-CH and β-CH_2_ protons reflected changes in the local electronic environment. Importantly, preservation of zein backbone resonances indicated site-selective conjugation without structural degradation, ensuring protein integrity while imparting active targeting functionality [23]. The disappearance of the crystalline peaks of TMZ and EA in the thermogram of FA–TMZ/EA PZ-CS NPs indicates successful encapsulation and amorphization of both drugs within the zein–PLGA matrix. The absence of melting endotherms demonstrates that the drugs are no longer in their native crystalline states but are molecularly dispersed or solubilized within the polymeric network, forming an amorphous or solid-solution phase. The broadening and downward shift of the PLGA glass transition suggest strong intermolecular interactions, particularly hydrogen bonding, between the drug molecules, polymer chains, and folic acid moieties on the nanoparticle surface. Such thermal modifications signify enhanced physical stability and homogeneity of the nanocarrier system. Moreover, the conversion of crystalline drugs into an amorphous state is advantageous for improving solubility and dissolution kinetics, which can contribute to enhanced bioavailability and synergistic therapeutic performance of TMZ and EA in glioblastoma therapy. Overall, the DSC results confirm the successful fabrication of a stable folic acid-functionalized zein–PLGA core–shell nanostructure with efficient dual drug entrapment and strong intermolecular compatibility among all components.

The XRD analysis provides crucial insight into the physical state and molecular dispersion of drugs within the nanoparticulate system. The disappearance of the characteristic crystalline peaks of TMZ, EA, and FA in the zein–PLGA and FA-zein–PLGA nanoparticles signifies a transition from crystalline to amorphous form during nanoparticle fabrication. This transformation is primarily attributed to intermolecular interactions (hydrogen bonding and hydrophobic interactions) between the drug molecules and polymeric carriers (PLGA and zein) as well as nano-confinement effects during solvent evaporation. The amorphous nature of the dual drug-loaded nanoparticles enhances the thermodynamic instability and molecular mobility of the encapsulated drugs, which in turn facilitates superior solubility and dissolution rate, critical for improving bioavailability and therapeutic efficacy against glioblastoma [24,25]. Furthermore, the absence of FA crystalline peaks after conjugation suggests that FA is successfully grafted onto the nanoparticle surface rather than existing as a separate crystalline phase, ensuring stable covalent linkage through EDC/NHS coupling [26].

The in vitro release profile confirms the successful fabrication of a hierarchical PLGA–zein core–shell nanostructure, in which the PLGA core acts as a hydrophobic reservoir for temozolomide (TMZ) while the outer zein shell governs ellagic acid (EA) diffusion, enabling sustained and synchronised dual-drug release. The limited initial burst arises from desorption of surface-associated drug, whereas prolonged release is controlled by diffusion through the polymeric matrix coupled with gradual PLGA erosion [27]. Release was significantly accelerated under acidic conditions due to proton-induced polymer relaxation, disruption of drug–zein interactions, and enhanced PLGA hydrolysis, promoting preferential drug liberation within the acidic tumour microenvironment while minimizing premature systemic release [28,29]. Kinetic modelling showed the best fit with the Korsmeyer–Peppas model for both drugs, indicating a diffusion–erosion–coupled mechanism typical of core–shell nanocarriers, with higher correlation at pH 5.5 supporting diffusion-dominated transport under acidic conditions. TMZ exhibited marginally higher correlation coefficients than EA, consistent with its smaller molecular size and higher diffusivity. The poor zero-order fit confirmed controlled, non-constant release. The coordinated co-release of TMZ and EA preserves nanocarrier integrity and is expected to enhance DNA damage-mediated apoptosis through PARP inhibition by EA, reinforcing the therapeutic synergy of this pH-responsive dual-drug platform [30]. FA-TMZ/EA-PZ-CS nanoparticles exhibited hemolysis values below the permissible limit (≤5%) across all evaluated concentrations, confirming their compatibility with red blood cells. Folic acid conjugation did not alter the haemolytic profile, indicating that covalent surface modification maintains blood safety. Additionally, the stable encapsulation of temozolomide and ellagic acid within distinct nanodomains minimizes premature drug exposure to blood components. These results demonstrate that the formulation meets essential hemocompatibility criteria for systemic administration and supports targeted glioblastoma therapy [31].

Biocompatibility is essential for the clinical translation of nanocarriers. The folic acid-conjugated PLGA–zein dual drug-loaded nanoparticles demonstrated excellent cytocompatibility, maintaining >80% viability in L929 fibroblasts across all tested concentrations (10–1000 µg/mL). L929 cells, recommended by ISO standards for cytotoxicity screening, provide a reliable model for assessing biomaterial safety. The preserved viability indicates minimal membrane damage, mitochondrial impairment, or metabolic stress in normal cells. This favourable profile is attributed to the biodegradable and FDA-approved nature of PLGA and the inherent biocompatibility of zein. Folic acid functionalization further enhances biological tolerance by reducing nonspecific cellular interactions and promoting preferential targeting toward folate receptor–overexpressing tumour cells. Moreover, the controlled release characteristic of the core–shell architecture limits burst drug exposure, thereby minimizing off-target toxicity. Overall, these findings confirm the high biosafety and cytocompatibility of the folate-targeted PLGA–zein nanosystem, supporting its further evaluation for targeted glioblastoma therapy [32,33,34].

The synergistic anticancer interaction between TMZ and EA in LN229 glioblastoma cells was quantitatively validated using the Chou–Talalay method implemented in CompuSyn software. While individual cytotoxicity profiling confirmed dose-dependent growth inhibition by both agents, combination treatment resulted in a markedly enhanced reduction in cell viability at lower effective doses. The calculated combination index (CI) values consistently below unity across multiple effect levels confirm true pharmacological synergism rather than additive or concentration-dependent toxicity [35,36]. This synergy is mechanistically attributable to PARP-mediated DNA repair inhibition by EA, which potentiates TMZ-induced DNA damage. TMZ exerts its cytotoxicity primarily through the formation of O^6^-methylguanine, leading to DNA strand breaks that are efficiently repaired by PARP-dependent base excision repair pathways. EA-mediated suppression of PARP activity compromises this repair machinery, resulting in the accumulation of unrepaired DNA lesions, replication stress, and enhanced apoptotic signalling. The observed dose-reduction effect further indicates that EA sensitizes glioblastoma cells to TMZ, enabling effective cytotoxicity at substantially lower TMZ concentrations an outcome with significant clinical relevance for minimizing systemic toxicity and resistance [5,6]. Importantly, LN229 cells are known for their intrinsic resistance to alkylating agents, underscoring the therapeutic relevance of this combination strategy. Collectively, the CompuSyn-based synergy analysis, together with PARP inhibition as a key molecular mechanism, supports TMZ–EA co-treatment as a rational strategy to overcome PARP -mediated chemoresistance in glioblastoma.

The MTT assay demonstrated that both TMZ/EA-PZ-CS NPs and FA-TMZ/EA-PZ-CS NPs elicited significantly enhanced cytotoxicity in LN229 glioblastoma cells compared with free drug combinations, confirming the therapeutic advantage of nanoencapsulation. The improved antiproliferative effect of the non-targeted core–shell nanoparticles can be attributed to efficient co-delivery, sustained intracellular drug release, and improved cellular internalization facilitated by the nanoscale architecture and protein–polymer hybrid matrix. Notably, folic acid-functionalized nanoparticles exhibited the greatest reduction in cell viability, indicating superior anticancer efficacy. This enhancement is primarily driven by folate receptor-mediated endocytosis, which promotes selective and increased intracellular accumulation of the nanoparticles in LN229 cells, known to overexpress folate receptors [37]. Enhanced intracellular delivery intensifies temozolomide-induced DNA alkylation while simultaneously amplifying ellagic acid-mediated PARP inhibition, resulting in impaired DNA repair, accumulation of lethal DNA damage, and amplified apoptotic signalling [38]. Furthermore, the reduced IC_50_ values observed for FA-TMZ/EA-PZ-CS NPs underscore a pronounced dose-sparing effect, highlighting the role of active targeting in overcoming intrinsic chemoresistance. Collectively, these findings confirm that folic acid-guided, dual drug-loaded core–shell nanoparticles offer a synergistic, targeted, and mechanistically reinforced strategy for enhancing temozolomide efficacy against resistant glioblastoma cells.

The enhanced cellular uptake of folic acid-conjugated PLGA–zein nanoparticles is attributed to folate receptor-α–mediated endocytosis, consistent with its overexpression in LN229 glioma cells. The markedly higher intracellular fluorescence relative to non-conjugated nanoparticles confirms the effectiveness of ligand-directed active targeting, while the limited uptake of non-targeted systems is likely due to nonspecific endocytic pathways. Predominant cytoplasmic localization indicates efficient intracellular trafficking and probable endosomal escape, which is essential for the therapeutic activity of temozolomide and ellagic acid. Overall, these results validate folate functionalization as a robust strategy to enhance glioma-specific uptake and intracellular drug delivery efficiency [14,39]. Although enhanced uptake and cytotoxicity of FA-conjugated nanoparticles were observed in LN229 cells, further validation using folate receptor-low/negative cell lines and competitive folic acid blocking studies would strengthen confirmation of receptor-mediated targeting.

The clonogenic assay provides a stringent measure of a cell’s capacity to undergo unlimited division and form macroscopic colonies, reflecting its long-term survival potential. The significant decline in surviving fraction observed with the dual drug-loaded nanosystems demonstrates that the combined action of TMZ and EA induces irreversible growth arrest and reproductive cell death in LN229 glioma cells. The superior inhibition achieved by the FA-conjugated nanoparticles can be attributed to enhanced cellular uptake through folate receptor-mediated endocytosis and improved intracellular retention of the therapeutics. The Trypan Blue assay demonstrated that folic acid-conjugated dual drug-loaded nanosystems significantly enhanced the cytotoxic efficacy against LN229 cells. The marked decline in cell viability in FA–TMZ + EA NPs compared to non-conjugated and control groups highlights the synergistic action of TMZ and EA, reinforced by folate receptor-mediated uptake. These findings confirm that active targeting via folic acid conjugation substantially improves nanoparticle internalization and therapeutic potency in glioma cells.

Hoechst 33342 staining confirmed apoptosis induction by the dual drug-loaded nanosystems. Non-conjugated TMZ/EA-PZ-CS NPs produced limited nuclear condensation, reflecting passive uptake and suboptimal intracellular drug accumulation. In contrast, FA-TMZ/EA-PZ-CS NPs induced pronounced chromatin condensation and nuclear fragmentation, affecting 60–75% of cells compared to 25–35% for non-targeted particles, highlighting the critical role of folate receptor-mediated targeting. The characteristic progression from karyopyknosis to karyorrhexis indicates activation of caspase-dependent apoptotic signalling. Caspase-mediated PARP cleavage suppresses DNA repair and facilitates irreversible commitment to apoptosis. The concordant nuclear disintegration and PARP inhibition observed in FA-targeted nanoparticles confirm the efficient induction of programmed cell death, demonstrating the therapeutic superiority of folic acid-guided dual drug delivery in glioma cells [40,41,42].

The AO/EB staining results clearly demonstrate that FA-TMZ/EA-PZ-CS NPs exhibit enhanced apoptotic activity compared to the non-conjugated counterpart and the untreated control. The transition from green to orange-red fluorescence corresponds to the progressive stages of apoptosis, confirming that the nanosystem triggers programmed cell death rather than necrosis. The improved apoptotic response in the FA-TMZ/EA-PZ-CS NPs can be attributed to folate receptor-mediated endocytosis, which enables preferential uptake by folate receptor-overexpressing LN229 glioma cells. This targeted mechanism enhances the intracellular concentration of the co-delivered drugs, TMZ and EA, leading to synergistic activation of apoptotic pathways. In glioblastoma cells, inhibition of PARP by ellagic acid suppresses the repair of temozolomide-induced DNA single-strand breaks, leading to replication fork collapse and accumulation of lethal double-strand DNA damage. The sustained DNA damage burden activates the ATM/ATR–p53 axis, promotes mitochondrial dysfunction through Bax/Bcl-2 imbalance, and culminates in caspase-dependent apoptosis. This mechanistic interplay explains the pronounced synergistic cytotoxicity observed for the TMZ–ellagic acid combination, particularly in DNA repair-compromised glioma cells. The combined effect leads to amplified PARP cleavage, chromatin condensation, and nuclear fragmentation, consistent with the observed fluorescence changes. These findings corroborate previous Hoechst staining results, indicating that the FA-guided nanosystem promotes controlled apoptosis through enhanced cellular uptake and targeted delivery [4,43].

The substantial PARP inhibition observed in LN229 cells, particularly with FA-TMZ/EA-PZ-CS NPs, provides quantitative protein-level validation of the synergistic potential of combining TMZ and EA within a targeted nano delivery platform. While Western blotting is frequently used for qualitative visualization, the use of a high-sensitivity ELISA in this study allows for the precise absolute quantification of PARP-1 protein concentrations (expressed in ng/mL). The observed reduction in protein expression (PARP inhibition) stems from a complementary mechanism: temozolomide induces DNA alkylation at the O^6^- and N^7^-guanine positions, recruiting PARP-1 to the damaged sites, while ellagic acid competitively inhibits PARP at the NAD^+^ catalytic domain. This simultaneous induction of DNA damage and PARP inhibition creates a synthetic lethal scenario through the “PARP trapping” phenomenon, where inhibited PARP–DNA complexes obstruct replication forks and transcription machinery, thereby overwhelming the DNA repair capacity. The lower IC_50_ value for FA-TMZ/EA-PZ-CS NPs suggests reduced therapeutic thresholds, potentially minimizing systemic toxicity while maximizing efficacy, critical for managing dose-limiting toxicities in glioblastoma treatment [44,45,46].

The qRT-PCR analysis demonstrates that nanoparticle-mediated co-delivery of temozolomide and ellagic acid significantly suppresses PARP1 expression in LN229 glioma cells. The significant reduction in PARP mRNA observed with non-targeted TMZ/EA-PZ-CS NPs can be attributed to sustained TMZ-induced DNA alkylation combined with EA-mediated PARP inhibition, which together generate persistent DNA damage and replication stress. The synergistic anticancer efficacy of TMZ and PARP inhibitors is driven by the coordinated induction of DNA damage and simultaneous suppression of DNA repair, culminating in synthetic lethality. TMZ exerts its cytotoxic effect by inducing DNA methylation at O^6^- and N^7^-guanine and N^3^-adenine residues, generating single-strand breaks (SSBs) during base excision repair (BER). Under physiological conditions, PARP-1 rapidly detects these lesions and orchestrates BER through NAD^+^-dependent poly(ADP-ribosyl)ation and recruitment of repair complexes. PARP inhibitors abrogate this protective response through dual mechanisms: catalytic inhibition of PARP activity and stabilization of PARP–DNA complexes, known as PARP trapping. Inhibition of PARP prevents resolution of TMZ-induced SSBs, which are subsequently converted into double-strand breaks (DSBs) during DNA replication. PARP trapping further obstructs replication fork progression and transcriptional machinery, intensifying replication stress and genomic instability. Persistent DNA damage activates ATM/ATR–CHK checkpoint signalling, driving prolonged cell-cycle arrest and transcriptional repression of DNA repair genes, including PARP1. This feedback-mediated downregulation further compromises repair capacity, reinforcing genotoxic stress. When damage surpasses the cellular repair threshold, apoptotic pathways are triggered through mitochondrial outer membrane permeabilization, cytochrome c release, and caspase-9/3 activation. Activated caspase-3 cleaves residual PARP protein, preventing energy-dependent repair and irreversibly committing cells to apoptosis. In conclusion, TMZ increases the DNA damage burden, whereas PARP inhibitors disable both functional and transcriptional DNA repair responses, promoting PARP trapping. This integrated mechanism converts repairable lesions into lethal DNA damage, effectively overcoming chemoresistance and enhancing apoptosis in glioblastoma cells. The downregulation of PARP expression achieved with FA-TMZ/EA-PZ-CS NPs highlights the role of active targeting in amplifying this genetic response. Folic acid-mediated receptor-dependent endocytosis enhances intracellular drug accumulation and retention, intensifying DNA damage signalling and promoting PARP trapping at DNA lesions. The accumulation of inhibited PARP–DNA complexes further reinforces feedback suppression of PARP-1 transcription. These results demonstrate that folate-guided nanodelivery augments both functional and transcriptional inhibition of PARP, thereby potentiating TMZ-induced cytotoxicity and contributing to the superior anticancer efficacy of the targeted nanosystem [5,47,48,49].

The comet assay revealed significantly enhanced DNA damage in LN229 glioblastoma cells treated with FA-TMZ/EA-PZ-CS NPs compared with non-targeted nanoparticles and controls, confirming the benefit of active targeting. Pronounced comet tail formation indicates extensive single- and double-strand DNA breaks. This effect arises from the complementary actions of the dual-drug system: TMZ induces DNA alkylation at O^6^- and N^7^-guanine residues, leading to replication fork stalling and the formation of double-strand breaks, while EA functionally suppresses base excision repair, promoting the persistence of unrepaired lesions. The PLGA–zein core–shell architecture enables sequential and sustained drug release, establishing a repair-deficient intracellular environment that amplifies cumulative DNA damage. This strategy is particularly effective in GBM, where enhanced DNA repair contributes to TMZ resistance, thereby underpinning the superior genotoxic efficacy of the targeted nanosystem [50,51].

The migratory capacity of glioma cells is a major contributor to tumour invasiveness and recurrence, making inhibition of migration a critical therapeutic goal. In the present study, the FA-TMZ/EA- PZ-CS NPs markedly suppressed LN229 cell migration compared to the non-targeted and untreated controls, highlighting the advantage of folate-mediated targeting and dual drug co-delivery. The enhanced anti-migratory activity of FA-TMZ/EA-PZ-CS NPs can be attributed to multiple synergistic mechanisms. Folic acid conjugation promotes receptor-mediated endocytosis, leading to increased intracellular accumulation of Temozolomide and Ellagic acid. Ellagic acid, acting as a natural PARP inhibitor, interferes with DNA repair and reduces expression of migration-related proteins (MMP-2, MMP-9), whereas temozolomide induces DNA alkylation and cytotoxic stress. Their co-delivery within the core–shell matrix ensures synchronized release, sustained intracellular exposure, and enhanced suppression of signalling pathways associated with cell motility and invasion [52]. These findings are consistent with previous reports that ligand-guided nanocarriers boosted cellular uptake and anti-migratory action in glioma models. Overall, the current findings highlight that FA-TMZ/EA-PZ-CS NPs not only improve drug delivery efficiency but also functionality limits glioma cell invasiveness, which is an important step towards limiting glioblastoma progression and recurrence.

Despite robust in vitro results, these findings face significant translational constraints. 2D LN229 monolayers fail to replicate the high interstitial fluid pressure (IFP), extracellular matrix (ECM) complexity, or the tumour heterogeneity characteristic of glioblastoma. While FA-functionalization facilitates receptor-mediated endocytosis, crossing the intact blood–brain barrier (BBB) remains a major physiological hurdle. However, the disorganized neovascularization of the blood–brain tumour barrier (BBTB) offers a “leaky” fenestrated endothelium that may enable passive accumulation via the enhanced permeability and retention (EPR) effect. This passive recruitment, coupled with active FA-ligand targeting, could improve therapeutic localization within the tumour core. Nonetheless, the current lack of in vivo pharmacokinetic (PK) and biodistribution data necessitates future validation in orthotopic xenograft models to rigorously assess site-specific accumulation and systemic safety. These results should therefore be interpreted as a foundational mechanistic proof-of-concept rather than a definitive translational profile.

## 5. Conclusions

This study reports the development of a folic acid-conjugated PLGA–zein core–shell nanosystem (FA-TMZ/EA-PZ-CS NPs) for the co-delivery of TMZ and EA, using folic acid as a targeting strategy to address chemoresistance in glioblastoma. The engineered core–shell architecture enabled efficient dual-drug encapsulation, physicochemical stability, and sustained, pH-responsive drug release. The key novelty of this work lies in the integration of chemotherapy with PARP-mediated DNA repair inhibition within a single ligand-targeted nanosystem. By incorporating EA as a natural PARP inhibitor alongside TMZ, the system is designed to enhance DNA damage while simultaneously suppressing repair pathways, thereby improving therapeutic efficacy. Furthermore, folic acid functionalization significantly enhanced cellular uptake in LN229 glioblastoma cells, resulting in superior cytotoxicity compared to non-targeted formulations. Mechanistic investigations demonstrated strong synergistic interaction between TMZ and EA, accompanied by enhanced apoptosis, inhibition of clonogenic survival and cell migration, and significant downregulation of PARP expression, confirming the role of EA in sensitizing tumour cells to TMZ-induced DNA damage. Despite these promising findings, the current study is limited to in vitro evaluation. Future studies will focus on in vivo pharmacokinetics, biodistribution, and therapeutic efficacy in orthotopic glioblastoma models to validate the translational potential of this nanosystem. Overall, this work presents a mechanistically driven, targeted nanotherapeutic strategy that combines dual-drug delivery with modulation of DNA repair pathways, offering a promising approach to overcoming chemoresistance in glioblastoma.

## Data Availability

The original contributions presented in this study are included in the article/Supplementary Material. Further inquiries can be directed to the corresponding author.

## Supplementary Materials

The following supporting information can be downloaded at: https://www.mdpi.com/article/10.3390/pharmaceutics18060655/s1.

## References

[1] Ou A., Alfred Yung W.K., Majd N. (2020). Molecular Mechanisms of Treatment Resistance in Glioblastoma. Int. J. Mol. Sci..

[2] Khaddour K., Johanns T.M., Ansstas G. (2020). The Landscape of Novel Therapeutics and Challenges in Glioblastoma Multiforme: Contemporary State and Future Directions. Pharmaceuticals.

[3] Rong L., Li N., Zhang Z. (2022). Emerging Therapies for Glioblastoma: Current State and Future Directions. J. Exp. Clin. Cancer Res..

[4] Tharamelveliyil Rajendran A., Dheeraj Rajesh G., Kumar P., Shivam Raju Dwivedi P., Shashidhara Shastry C., Narayanan Vadakkepushpakath A. (2023). Selection of Potential Natural Compounds for Poly-ADP-Ribose Polymerase (PARP) Inhibition in Glioblastoma Therapy by in Silico Screening Methods. Saudi J. Biol. Sci..

[5] Bisht P., Kumar V.U., Pandey R., Velayutham R., Kumar N. (2022). Role of PARP Inhibitors in Glioblastoma and Perceiving Challenges as Well as Strategies for Successful Clinical Development. Front. Pharmacol..

[6] Cella E., Bosio A., Persico P., Caccese M., Padovan M., Losurdo A., Maccari M., Cerretti G., Ius T., Minniti G. (2024). PARP Inhibitors in Gliomas: Mechanisms of Action, Current Trends and Future Perspectives. Cancer Treat. Rev..

[7] Parrish K.E., Cen L., Murray J., Calligaris D., Kizilbash S., Mittapalli R.K., Carlson B.L., Schroeder M.A., Sludden J., Boddy A.V. (2015). Efficacy of PARP Inhibitor Rucaparib in Orthotopic Glioblastoma Xenografts Is Limited by Ineffective Drug Penetration into the Central Nervous System. Mol. Cancer Ther..

[8] Lemasson B., Wang H., Galbán S., Li Y., Zhu Y., Heist K.A., Tsein C., Chenevert T.L., Rehemtulla A., Galbán C.J. (2016). Evaluation of Concurrent Radiation, Temozolomide and ABT-888 Treatment Followed by Maintenance Therapy with Temozolomide and ABT-888 in a Genetically Engineered Glioblastoma Mouse Model. Neoplasia.

[9] Sargazi S., Mukhtar M., Rahdar A., Barani M., Pandey S., Díez-Pascual A.M. (2021). Active Targeted Nanoparticles for Delivery of Poly(ADP-Ribose) Polymerase (PARP) Inhibitors: A Preliminary Review. Int. J. Mol. Sci..

[10] Ceci C., Lacal P.M., Tentori L., De Martino M.G., Miano R., Graziani G. (2018). Experimental Evidence of the Antitumor, Antimetastatic and Antiangiogenic Activity of Ellagic Acid. Nutrients.

[11] Jenjob R., Phakkeeree T., Crespy D. (2020). Core–Shell Particles for Drug-Delivery, Bioimaging, Sensing, and Tissue Engineering. Biomater. Sci..

[12] Lu B., Lv X., Le Y. (2019). Chitosan-Modified PLGA Nanoparticles for Control-Released Drug Delivery. Polymers.

[13] Yu X., Wu H., Hu H., Dong Z., Dang Y., Qi Q., Wang Y., Du S., Lu Y. (2020). Zein Nanoparticles as Nontoxic Delivery System for Maytansine in the Treatment of Non-Small Cell Lung Cancer. Drug Deliv..

[14] Pourmasoumi P., Abdouss M., Farhadi M., Jameie S.B., Khonakdar H.A. (2024). Co-Delivery of Temozolomide and Quercetin with Folic Acid-Conjugated Exosomes in Glioblastoma Treatment. Nanomedicine.

[15] Ghaly H.S.A., Seyedasli N., Varamini P. (2025). Enhanced Nanoprecipitation Method for the Production of PLGA Nanoparticles for Oncology Applications. AAPS J..

[16] Narayanan S., Pavithran M., Viswanath A., Narayanan D., Mohan C.C., Manzoor K., Menon D. (2014). Sequentially Releasing Dual-Drug-Loaded PLGA–Casein Core/Shell Nanomedicine: Design, Synthesis, Biocompatibility and Pharmacokinetics. Acta Biomater..

[17] Feng Y., Liao Z., Li M., Zhang H., Li T., Qin X., Li S., Wu C., You F., Liao X. (2023). Mesoporous Silica Nanoparticles-Based Nanoplatforms: Basic Construction, Current State, and Emerging Applications in Anticancer Therapeutics. Adv. Healthc. Mater..

[18] Lee C.Y., Ooi H., Grumezescu A.M., Torchilin V.P. (2016). Preparation of Temozolomide-Loaded Nanoparticles for Glioblastoma Multiforme Targeting—Ideal Versus Reality. Pharmaceuticals.

[19] Saha C., Kaushik A., Das A., Pal S., Majumder D. (2016). Anthracycline Drugs on Modified Surface of Quercetin-Loaded Polymer Nanoparticles: A Dual Drug Delivery Model for Cancer Treatment. PLoS ONE.

[20] Kim S.M., Patel M., Patel R. (2021). PLGA Core-Shell Nano/Microparticle Delivery System for Biomedical Application. Polymers.

[21] Pourhossein A., Rezaei S., Shojaosadati S.A. (2025). Folate-Functionalized Zein-PEI Nanoparticles for Targeted Cancer Therapy: Integrated Experimental and Molecular Dynamics Insights into Stability, Uptake, and Cytotoxicity. Int. J. Biol. Macromol..

[22] Öztekin M., Açıkel Y.S. (2025). Novel PH-Responsive Folic Acid-Conjugated Zein-Carboxymethyl Cellulose Nanoparticles for Enhanced and Controlled Curcumin Release. J. Pharm. Innov..

[23] Chuacharoen T., Sabliov C.M. (2017). Zein Nanoparticles as Delivery Systems for Covalently Linked and Physically Entrapped Folic Acid. J. Nanopart. Res..

[24] Singh G., Faruk A., Bedi P.M.S. (2018). Spectral Analysis of Drug Loaded Nanoparticles for Drug-Polymer Interactions. J. Drug Deliv. Ther..

[25] Kumar M. (2022). XRD Analysis for Characterization of Green Nanoparticles: A Mini Review. Glob. J. Pharm. Pharm. Sci..

[26] Sarnaik S., Ahmed H., Hussain N., Kundu S., Sahu B.D., Alexander A. (2025). Folic Acid-Chitosan Conjugated Mesoporous Silica Nanoparticles for Enhanced Piceatannol Uptake in MCF-7 Breast Cancer Cells. ACS Omega.

[27] Ananta J.S., Paulmurugan R., Massoud T.F. (2016). Temozolomide-Loaded PLGA Nanoparticles to Treat Glioblastoma Cells: A Biophysical and Cell Culture Evaluation. Neurol. Res..

[28] Annappa H., Tamatam A., Nallamuthu I., Ranganathan K. (2025). Formulation of PH-Responsive Nanoparticles Using Zein/Sodium Alginate Polymers for Enhanced Bioavailability of the Vitamin D3. Int. J. Biol. Macromol..

[29] Kaushik P., Priyadarshini E., Rawat K., Rajamani P., Bohidar H.B. (2020). PH Responsive Doxorubucin Loaded Zein Nanoparticle Crosslinked Pectin Hydrogel as Effective Site-Specific Anticancer Substrates. Int. J. Biol. Macromol..

[30] Zhu W., Long J., Shi M. (2023). Release Kinetics Model Fitting of Drugs with Different Structures from Viscose Fabric. Materials.

[31] De La Harpe K.M., Kondiah P.P.D., Choonara Y.E., Marimuthu T., Du Toit L.C., Pillay V. (2019). The Hemocompatibility of Nanoparticles: A Review of Cell–Nanoparticle Interactions and Hemostasis. Cells.

[32] Witika B.A., Makoni P.A., Matafwali S.K., Chabalenge B., Mwila C., Kalungia A.C., Nkanga C.I., Bapolisi A.M., Walker R.B. (2020). Biocompatibility of Biomaterials for Nanoencapsulation: Current Approaches. Nanomaterials.

[33] Naahidi S., Jafari M., Edalat F., Raymond K., Khademhosseini A., Chen P. (2013). Biocompatibility of Engineered Nanoparticles for Drug Delivery. J. Control. Release.

[34] Kyriakides T.R., Raj A., Tseng T.H., Xiao H., Nguyen R., Mohammed F.S., Halder S., Xu M., Wu M.J., Bao S. (2021). Biocompatibility of Nanomaterials and Their Immunological Properties. Biomed. Mater..

[35] Pellosi D.S., Paula L.B., De Melo M.T., Tedesco A.C. (2019). Targeted and Synergic Glioblastoma Treatment: Multifunctional Nanoparticles Delivering Verteporfin as Adjuvant Therapy for Temozolomide Chemotherapy. Mol. Pharm..

[36] Zhao M., Bozzato E., Joudiou N., Ghiassinejad S., Danhier F., Gallez B., Préat V. (2019). Codelivery of Paclitaxel and Temozolomide through a Photopolymerizable Hydrogel Prevents Glioblastoma Recurrence after Surgical Resection. J. Control. Release.

[37] Ramalho M.J., Alves B., Andrade S., Lima J., Loureiro J.A., Pereira M.C. (2024). Folic-Acid-Conjugated Poly (Lactic-Co-Glycolic Acid) Nanoparticles Loaded with Gallic Acid Induce Glioblastoma Cell Death by Reactive-Oxygen-Species-Induced Stress. Polymers.

[38] CETIN A., BILTEKIN B. (2020). Ellagic Acid Enhances Antitumor Efficacy of Temozolomide in an in Vitro Glioblastoma Model. Turk. Neurosurg..

[39] McCord E., Pawar S., Koneru T., Tatiparti K., Sau S., Iyer A.K. (2021). Folate Receptors’ Expression in Gliomas May Possess Potential Nanoparticle-Based Drug Delivery Opportunities. ACS Omega.

[40] Mashimo M., Onishi M., Uno A., Tanimichi A., Nobeyama A., Mori M., Yamada S., Negi S., Bu X., Kato J. (2020). The 89-KDa PARP1 Cleavage Fragment Serves as a Cytoplasmic PAR Carrier to Induce AIF-Mediated Apoptosis. J. Biol. Chem..

[41] Los M., Mozoluk M., Ferrari D., Stepczynska A., Stroh C., Renz A., Herceg Z., Wang Z.Q., Schulze-Osthoff K. (2002). Activation and Caspase-Mediated Inhibition of PARP: A Molecular Switch between Fibroblast Necrosis and Apoptosis in Death Receptor Signaling. Mol. Biol. Cell.

[42] Song J., Cheng M., Xie Y., Li K., Zang X. (2023). Efficient Tumor Synergistic Chemoimmunotherapy by Self-Augmented ROS-Responsive Immunomodulatory Polymeric Nanodrug. J. Nanobiotechnol..

[43] Higuchi F., Nagashima H., Ning J., Koerner M.V.A., Wakimoto H., Cahill D.P. (2020). Restoration of Temozolomide Sensitivity by PARP Inhibitors in Mismatch Repair Deficient Glioblastoma Is Independent of Base Excision Repair. Clin. Cancer Res..

[44] Montaldi A.P., Lima S.C.G., Godoy P.R.D.V., Xavier D.J., Sakamoto-Hojo E.T. (2020). PARP-1 Inhibition Sensitizes Temozolomide-treated Glioblastoma Cell Lines and Decreases Drug Resistance Independent of MGMT Activity and PTEN Proficiency. Oncol. Rep..

[45] Jones A.B., Tuy K., Hawkins C.C., Quinn C.H., Saad J., Gary S.E., Beierle E.A., Ding L., Rochlin K.M., Lamb L.S. (2024). Temozolomide and the PARP Inhibitor Niraparib Enhance Expression of Natural Killer Group 2D Ligand ULBP1 and Gamma-Delta T Cell Cytotoxicity in Glioblastoma. Cancers.

[46] Tharamelveliyil Rajendran A., Dheeraj Rajesh G., Ashtekar H., Sairam A., Kumar P., Vadakkepushpakath A.N. (2024). Uncovering Naringin’s Anticancer Mechanisms in Glioblastoma via Molecular Docking and Network Pharmacology Approaches. Sci. Rep..

[47] Wu S., Li X., Gao F., De Groot J.F., Koul D., Yung W.K.A. (2021). PARP-Mediated PARylation of MGMT Is Critical to Promote Repair of Temozolomide-Induced O6-Methylguanine DNA Damage in Glioblastoma. Neuro. Oncol..

[48] Gill S.J., Travers J., Pshenichnaya I., Kogera F.A., Barthorpe S., Mironenko T., Richardson L., Benes C.H., Stratton M.R., McDermott U. (2015). Combinations of PARP Inhibitors with Temozolomide Drive PARP1 Trapping and Apoptosis in Ewing’s Sarcoma. PLoS ONE.

[49] Rose M., Burgess J.T., O’Byrne K., Richard D.J., Bolderson E. (2020). PARP Inhibitors: Clinical Relevance, Mechanisms of Action and Tumor Resistance. Front. Cell Dev. Biol..

[50] Wang Y., Luo W., Wang Y. (2019). PARP-1 and Its Associated Nucleases in DNA Damage Response. DNA Repair.

[51] Paradkar S., Purcell J., Cui A., Friedman S., Noronha K.J., Murray M.A., Sundaram R.K., Bindra R.S., Jensen R.B. (2024). PARG Inhibition Induces Nuclear Aggregation of PARylated PARP1. Structure.

[52] Dongliang W., Chen Q., Tan Y., Liu B., Liu C. (2017). Ellagic Acid Inhibits Human Glioblastoma Growth in Vitro and in Vivo. Oncol. Rep..

