# Supplementary material for: Folic Acid-Guided PLGA-Zein Core–Shell Nanoparticles for Co-Delivery of Temozolomide and Ellagic Acid to Overcome PARP-Mediated Chemoresistance in Glioblastoma
Source: Pharmaceutics. 2026 May 27;18(6):655. doi: 10.3390/pharmaceutics18060655 (PMC13304799; doi:10.3390/pharmaceutics18060655)
Supplement: Supplementary file 1 [file pharmaceutics-18-00655-s001.zip › pharmaceutics-4254880-supplementary.pdf]

Folic Acid-Guided Zein-PLGA Core-Shell Nanoparticles for Co-Delivery of Temozolomide and Ellagic Acid to Overcome PARP-Mediated Chemoresistance in Glioblastoma

Supplementary Data

Table S1: Summary of particle size, polydispersity index (PDI), zeta potential, drug loading content, and entrapment efficiency of developed PLGA-based nanoparticle formulations.

Sl No.	Nanosystems	Particle size (nm)	Zeta potential (mV)	PDI	Entrapment efficiency (%)	Drug loading content
1	Plain PLGA NPs	67.30	-28.2	0.140	-NA-	-NA
2	PLGA-Zein Core shell NPs	91.5	-38.0	0.162	-NA-	-NA-
3	TMZ/EA-PZ-CS NPs	190	-30.5	0.141	TMZ:94.68<br>EA: 93.71	TMZ:23.7<br>EA: 23.4
4	FA-TMZ/EA-PZ CS NPs	206	-32.5	0.118	-	-

Table S2: Kinetic modelling of TMZ/EA-PZ-CS NPs

TMZ/EA-PZ-CS NPs				
**pH**	**Zero-order R^2**	**First-order R^2**	**Higuchi R^2**	**Korsmeyer-Peppas R^2**
TMZ 7.4	0.9023	0.9614	0.9837	0.9926
EA 7.4	0.8251	0.9362	0.9668	0.9941
TMZ 5.5	0.8459	0.9567	0.9696	0.9974
EA 5.5	0.7483	0.9086	0.9272	0.9982

S1. Particle size distribution and Zeta potential of FA-TMZ/EA PZ CS NPs

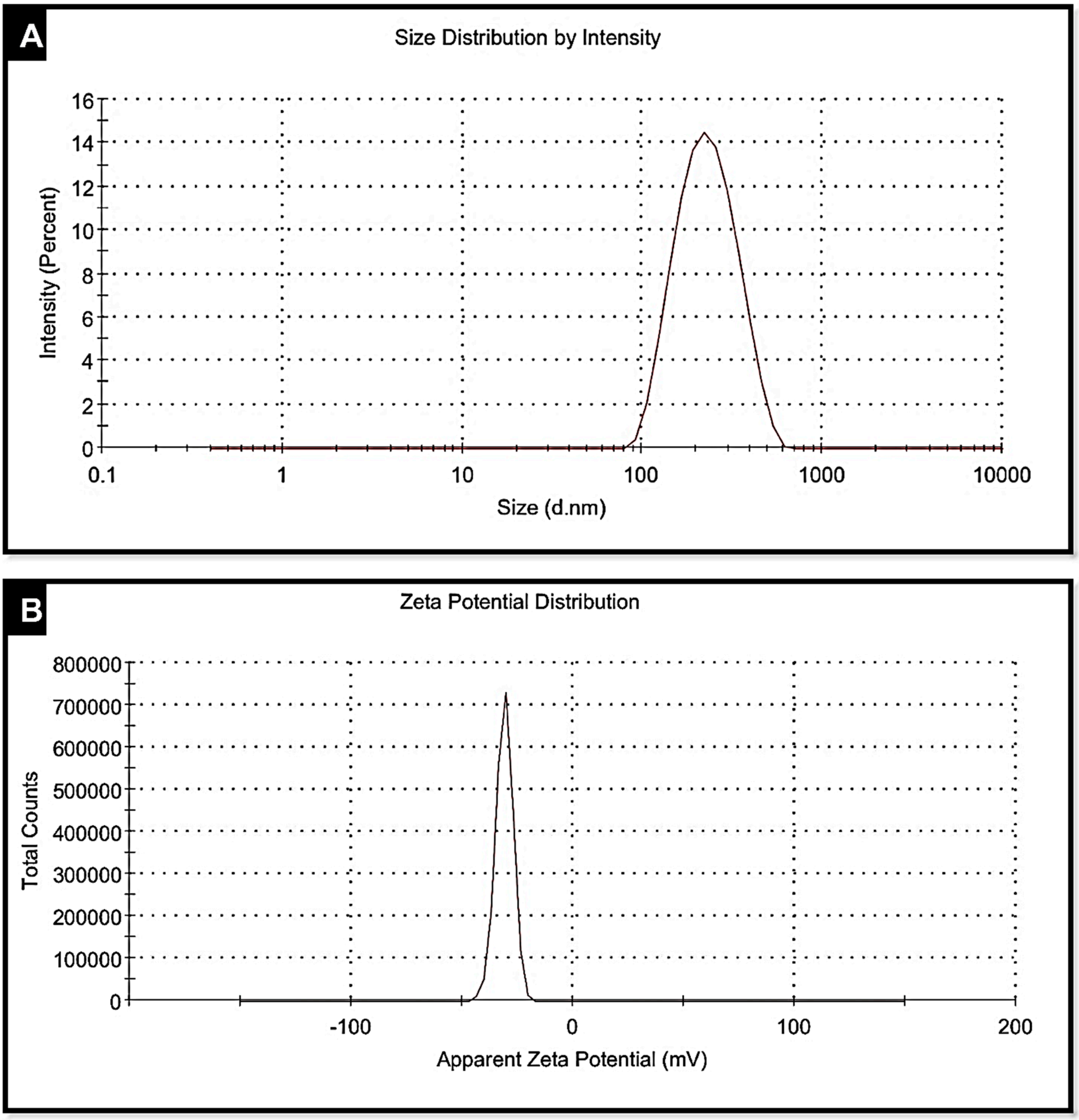

Figure S1. DLS and zeta potential pattern of FA-TMZ/EA-PZ-CS NPs

S2 . UV-Visible spectra of Temozolomide and Ellagic acid

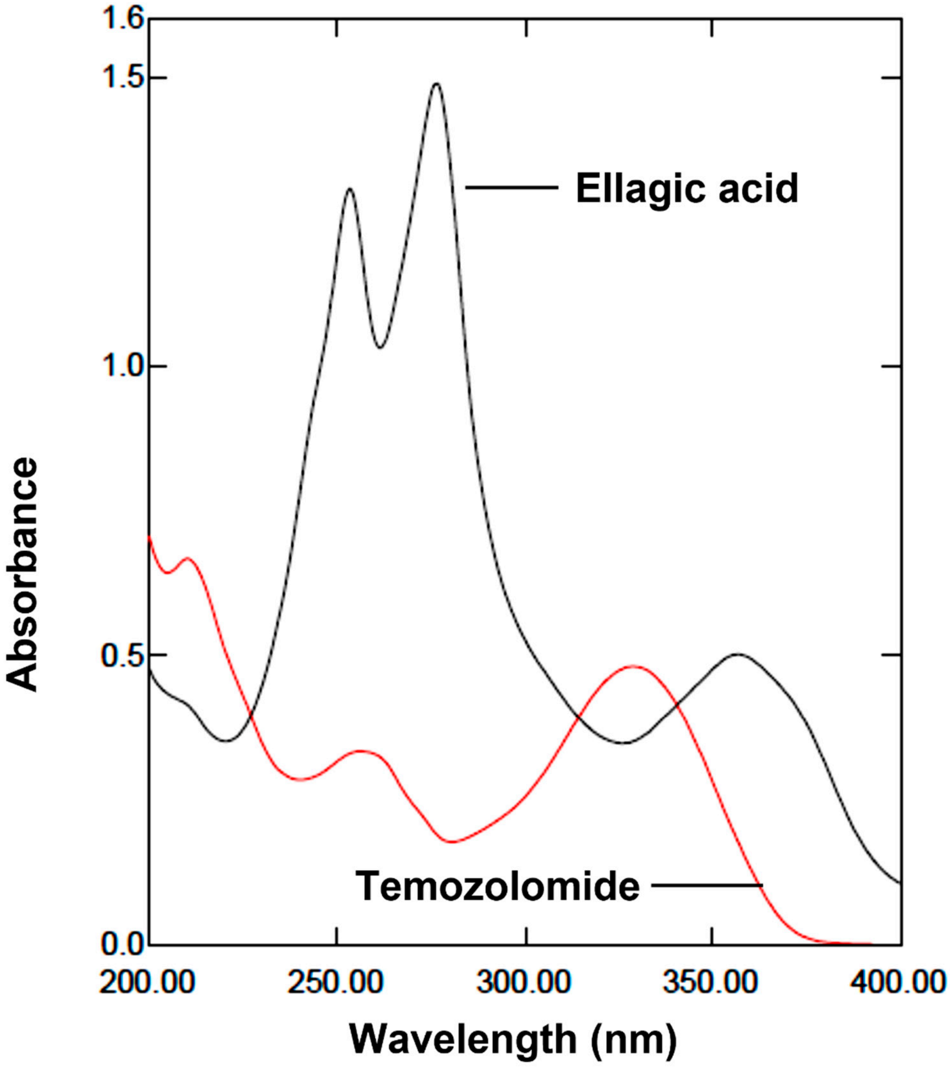

Figure S2. UV Absorption Spectra of Temozolomide and Ellagic Acid for λmax Determination

S3. Calibration curve of Ellagic acid and Temozolomide

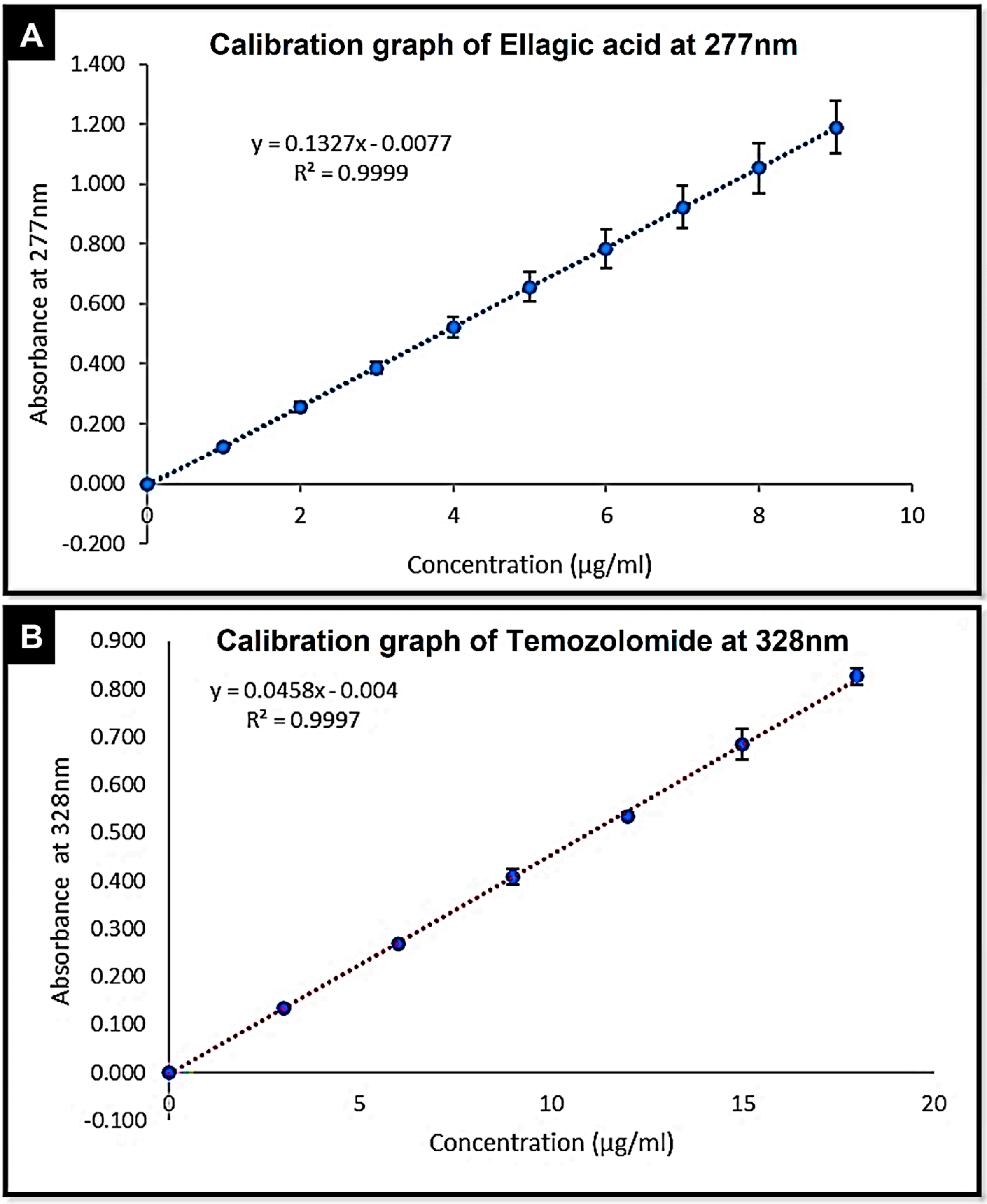

Figure S3. Calibration Curves of Ellagic Acid and Temozolomide by UV–Visible Spectroscopy

S4. FT-IR spectra of A) Zein, B) PLGA, C) Temozolomide, D) Ellagic acid, and E) Folic acid

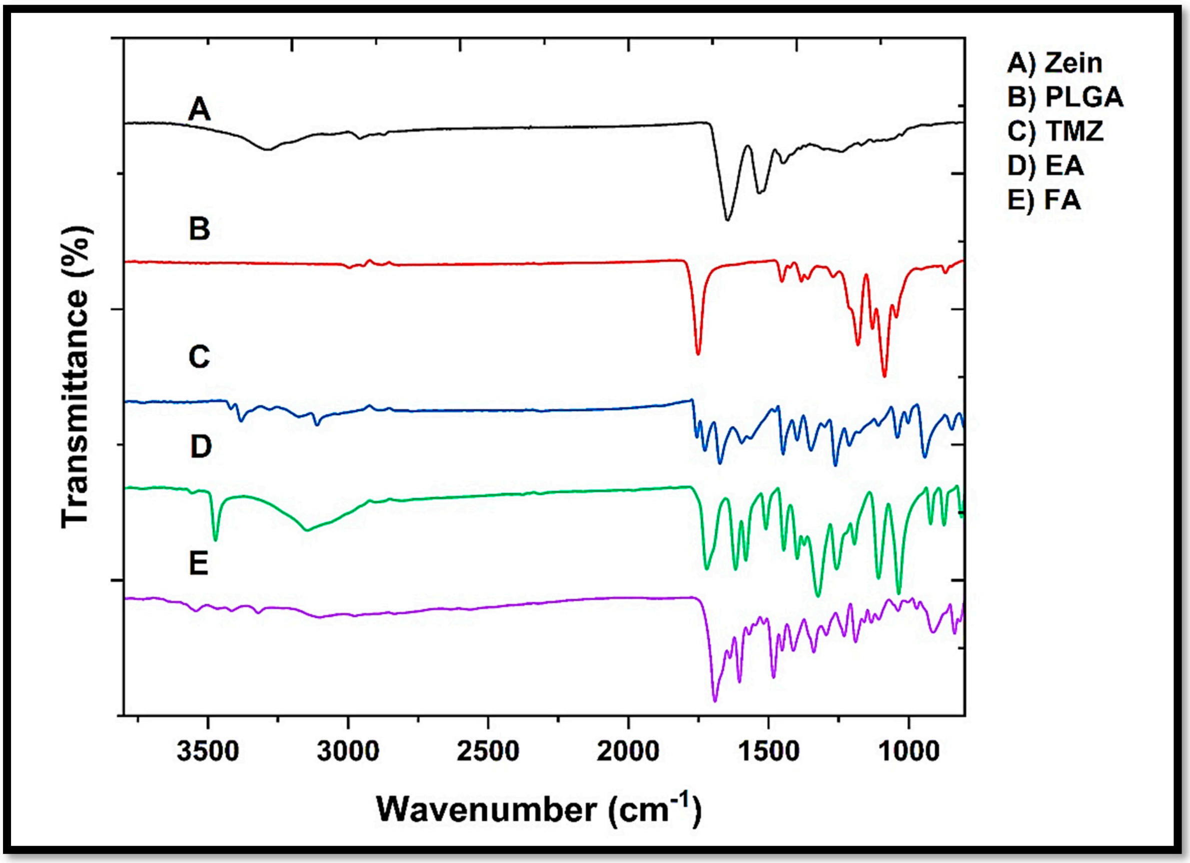

Figure S4. FT-IR spectra of A) Zein, B) PLGA, C) Temozolomide, D) Ellagic acid, and E) Folic acid

S5. ^{1}H NMR spectra of (A) Zein, (B) Folic acid, (C) Folic acid-Zein NPs (D) EDC/NHS mechanism of folic acid conjugation on zein NPs.

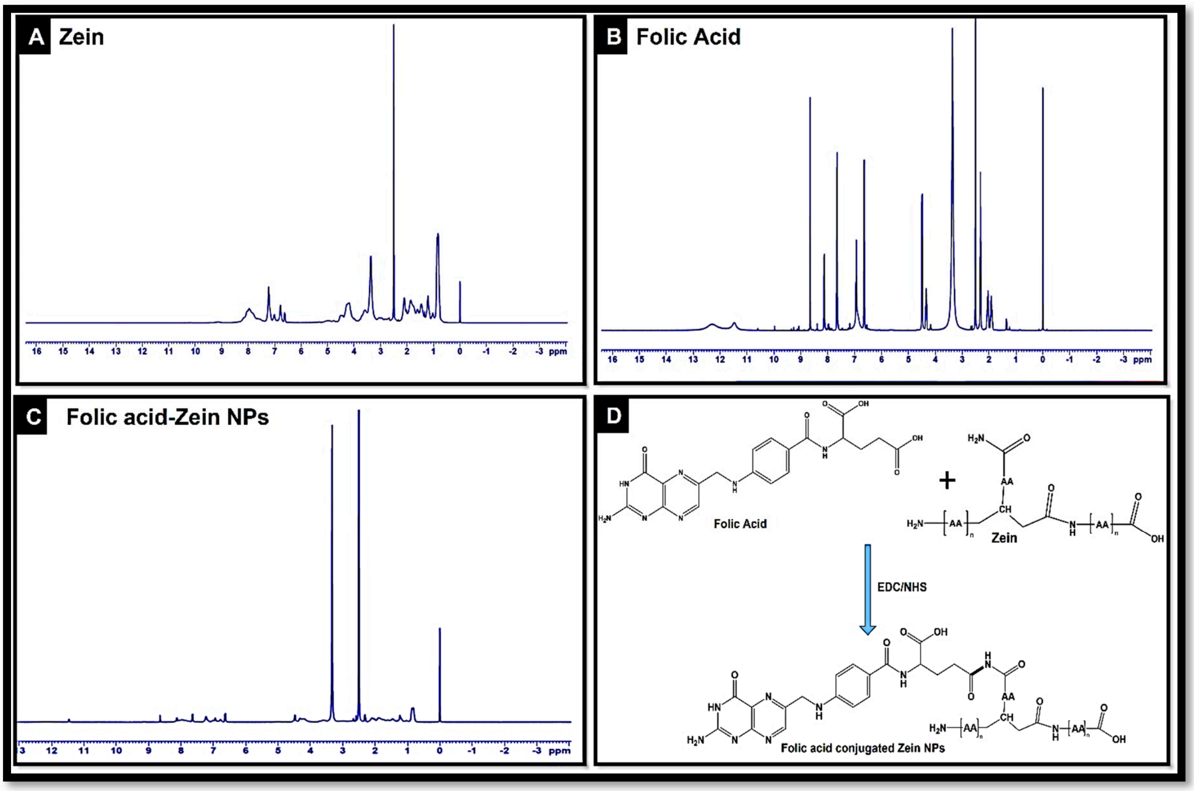

Figure S5. ^{1}H NMR spectra of (A) Zein, (B) Folic acid, (C) Folic acid-Zein NPs (D) EDC/NHS mechanism of folic acid conjugation on zein NPs.

S6. Differential scanning calorimetry thermograms of (A) Zein, (B) PLGA (75:25), (C) Temozolomide, (D) Ellagic acid, (E) Folic acid (F) FA-TMZ/EA-PZ-CS NPs.

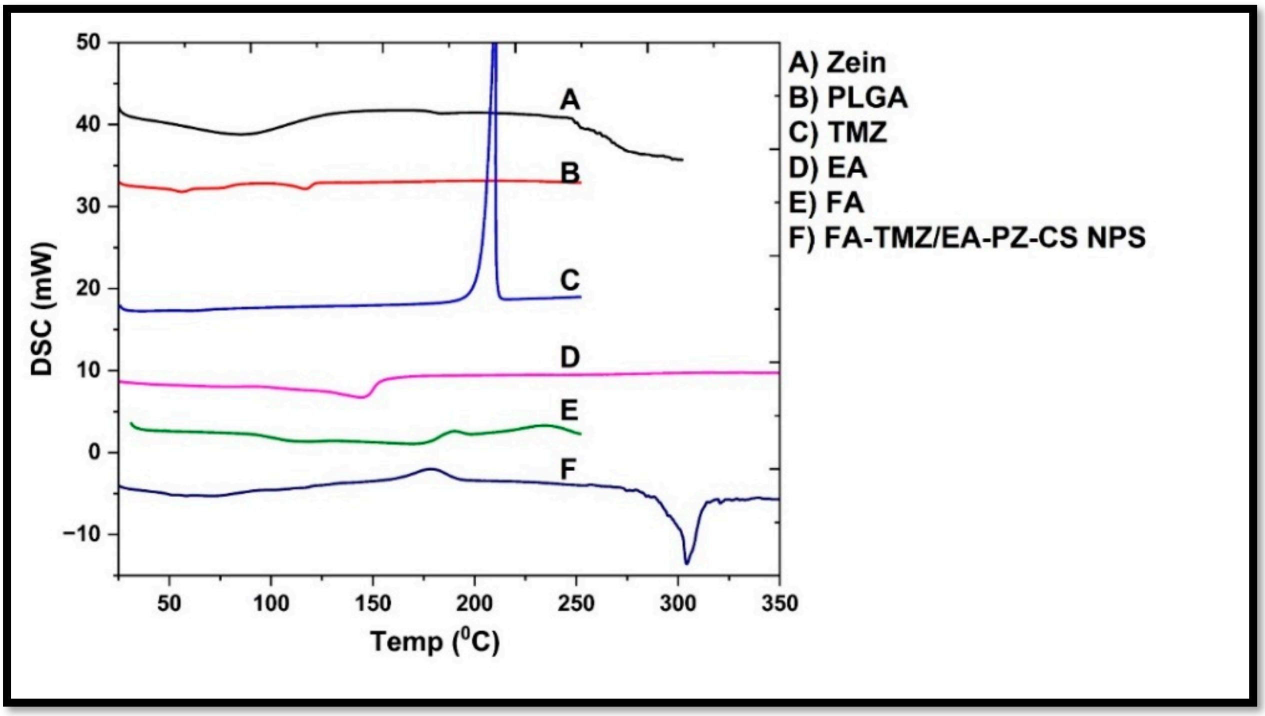

Figure S6. Differential scanning calorimetry thermograms of (A) Zein, (B) PLGA (75:25), (C) Temozolomide, (D) Ellagic acid, (E) Folic acid (F) FA-TMZ/EA-PZ-CS NPs. Heating rate: 10°C/min; nitrogen atmosphere; temperature range: 25-400°C.

S7. X-ray diffraction patterns of (a) Zein, (b) PLGA (c) Temozolomide (d) Ellagic acid, (e) Folic acid, (f) blank zein-PLGA core-shell nanoparticles, (g) folic acid-conjugated dual drug-loaded core-shell nanoparticles (FA-TMZ/EA-PZ-CS NPs).

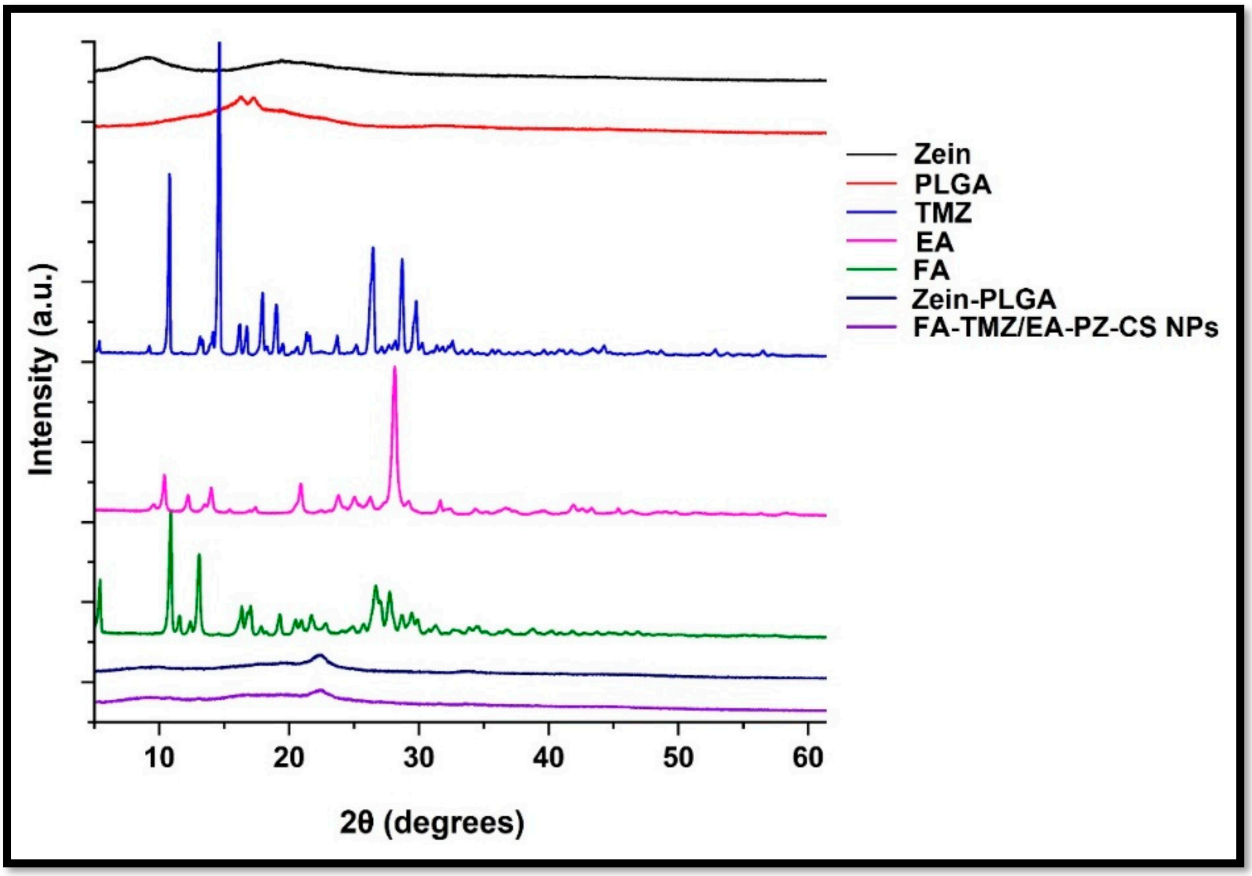

Figure S7. X-ray diffraction patterns of (a) Zein, (b) PLGA (c) Temozolomide (d) Ellagic acid, (e) Folic acid, (f) blank zein-PLGA core-shell nanoparticles, (g) folic acid-conjugated dual drug-loaded core-shell nanoparticles (FA-TMZ/EA-PZ-CS NPs).

S8. In vitro cumulative release profile of TMZ and EA from TMZ/EA-PZ-CS NPs at pH 7.4 and pH 5.5.

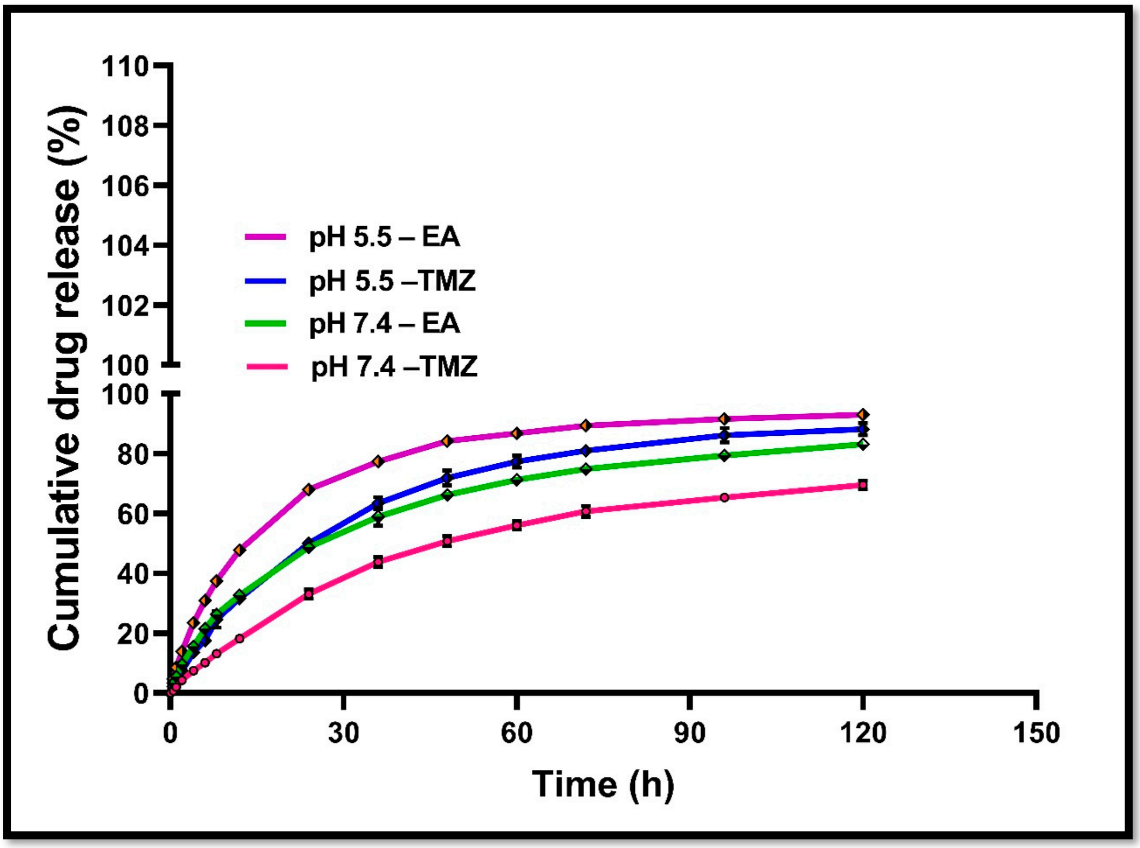

Figure S8. **In vitro cumulative release profile of TMZ and EA from TMZ/EA-PZ-CS NPs at pH 7.4 and pH 5.5. Data expressed as mean ± SD (n=3)**

S9. Hemocompatibility of FA-TMZ/EA-PZ-CS NPs

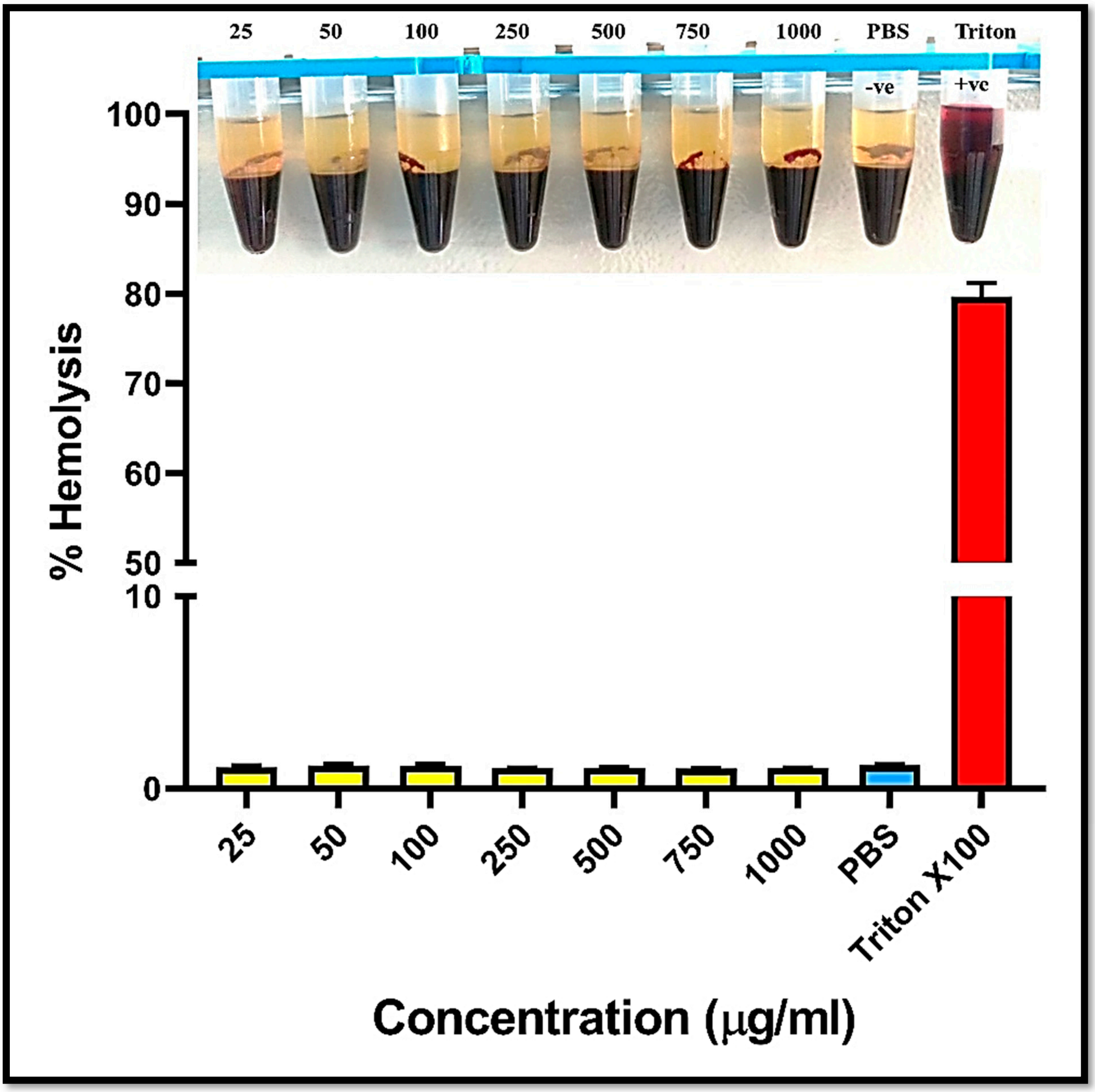

Figure S9. Represents the hemocompatibility results of FA-TMZ/EA-PZ-CS NPs using the hemolysis assay.

S10. cytocompatibility analysis results of FA-TMZ/EA-PZ-CS NPs

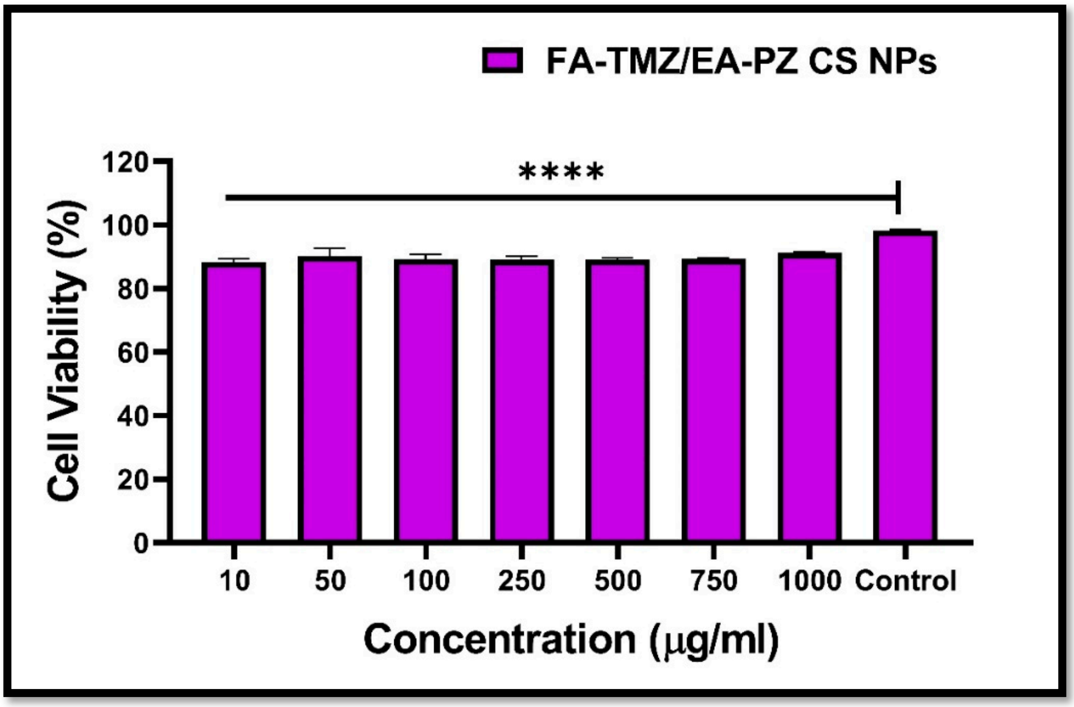

Figure S10. Represents the cytocompatibility analysis results of FA-TMZ/EA-PZ-CS NPs using the MTT assay in L929 cells

S11. The synergistic effect of TMZ and EA on the viability of LN229 GBM cells.

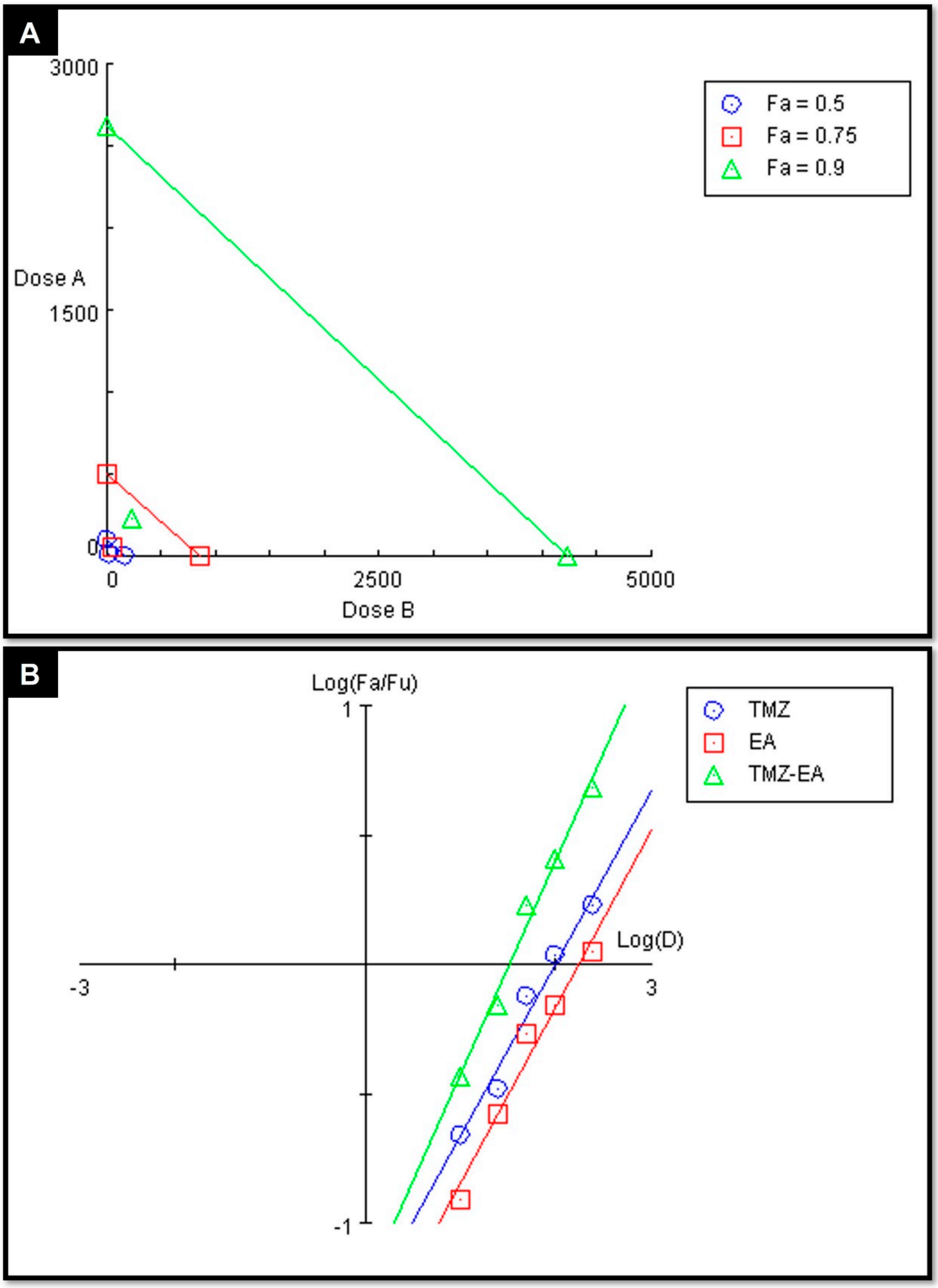

Figure S11. The synergistic effect of TMZ and EA on the viability of LN229 GBM cells. Estimation of the effect of drug combination using CompuSyn software: A) Isobologram, B) Median-effect plot. Data are presented as mean ± SD (n = 3 independent experiments, each performed in triplicate wells).

S12. Trypan blue dye exclusion assay

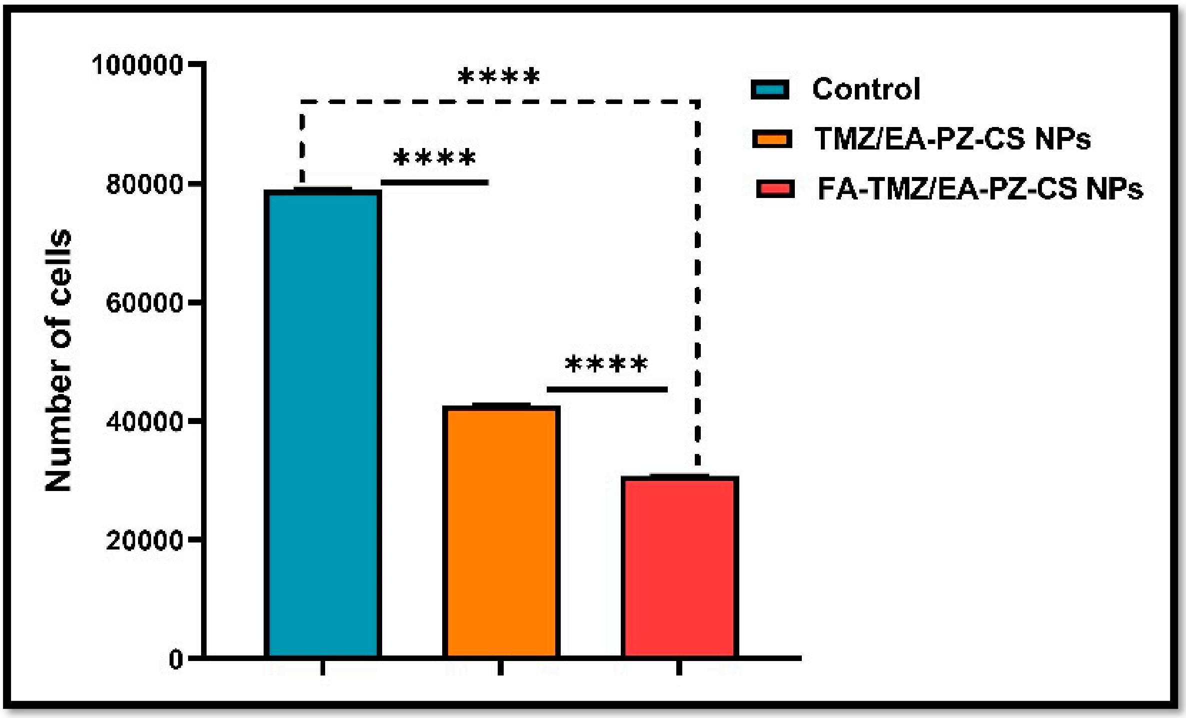

Figure S12. Quantification of viable LN229 cells using the Trypan Blue dye exclusion assay following treatment with control, TMZ/EA-PZ-CS NPs, and FA-TMZ/EA-PZ-CS NPs. One-way ANOVA followed by Tukey's Post hoc test $p<0.0001$ (n=3)
